# Evidence for a novel overlapping coding sequence in *POLG* initiated at a CUG start codon

**DOI:** 10.1186/s12863-020-0828-7

**Published:** 2020-03-06

**Authors:** Yousuf A. Khan, Irwin Jungreis, James C. Wright, Jonathan M. Mudge, Jyoti S. Choudhary, Andrew E. Firth, Manolis Kellis

**Affiliations:** 1 Department of Molecular and Cellular Physiology, Stanford University School of Medicine, Stanford, CA 94305 USA; 2 Division of Virology, Department of Pathology, University of Cambridge, Tennis Court Road, Cambridge, CB2 1QP UK; 3 Computer Science and Artificial Intelligence Laboratory, Massachusetts Institute of Technology, Cambridge, MA 02139 USA; 4 Broad Institute of MIT and Harvard, Cambridge, MA 02142 USA; 5 Functional Proteomics, Division of Cancer Biology, Institute of Cancer Research, 123 Old Brompton Road, London, SW7 3RP UK; 6 European Molecular Biology Laboratory, European Bioinformatics Institute, Wellcome Genome Campus, Hinxton, Cambridge, CB10 1SD UK

**Keywords:** POLG, CUG, Initiation, Ribosome, Polymerase, Mitochondria, Synonymous site conservation, synplot2, PhyloCSF

## Abstract

**Background:**

*POLG*, located on nuclear chromosome 15, encodes the DNA polymerase γ(Pol γ). Pol γ is responsible for the replication and repair of mitochondrial DNA (mtDNA). Pol γ is the only DNA polymerase found in mitochondria for most animal cells. Mutations in *POLG* are the most common single-gene cause of diseases of mitochondria and have been mapped over the coding region of the *POLG* ORF.

**Results:**

Using PhyloCSF to survey alternative reading frames, we found a conserved coding signature in an alternative frame in exons 2 and 3 of *POLG*, herein referred to as ORF-Y that arose de novo in placental mammals. Using the synplot2 program, synonymous site conservation was found among mammals in the region of the *POLG* ORF that is overlapped by ORF-Y. Ribosome profiling data revealed that ORF-Y is translated and that initiation likely occurs at a CUG codon. Inspection of an alignment of mammalian sequences containing ORF-Y revealed that the CUG codon has a strong initiation context and that a well-conserved predicted RNA stem-loop begins 14 nucleotides downstream. Such features are associated with enhanced initiation at near-cognate non-AUG codons. Reanalysis of the Kim et al. (2014) draft human proteome dataset yielded two unique peptides that map unambiguously to ORF-Y. An additional conserved uORF, herein referred to as ORF-Z, was also found in exon 2 of *POLG*. Lastly, we surveyed Clinvar variants that are synonymous with respect to the *POLG* ORF and found that most of these variants cause amino acid changes in ORF-Y or ORF-Z.

**Conclusions:**

We provide evidence for a novel coding sequence, ORF-Y, that overlaps the POLG ORF. Ribosome profiling and mass spectrometry data show that ORF-Y is expressed. PhyloCSF and synplot2 analysis show that ORF-Y is subject to strong purifying selection. An abundance of disease-correlated mutations that map to exons 2 and 3 of *POLG* but also affect ORF-Y provides potential clinical significance to this finding.

## Background

Mitochondria provide the majority of ATP for most cells. Mitochondria generate ATP via the electron transport chain (ETC) [1]. A number of ETC proteins are translated from mRNAs transcribed from genes in the mitochondrial DNA (mtDNA). The mitochondrial genome in humans is a circular DNA that encodes 13 proteins related to the function of the ETC, 22 tRNAs, and 2 rRNAs [2]. mtDNA is replicated by a complex of Pol γ, a ssDNA binding protein, the Twinkle mtDNA helicase, topoisomerases, and RNaseH activity [3].

**Fig. 1:**
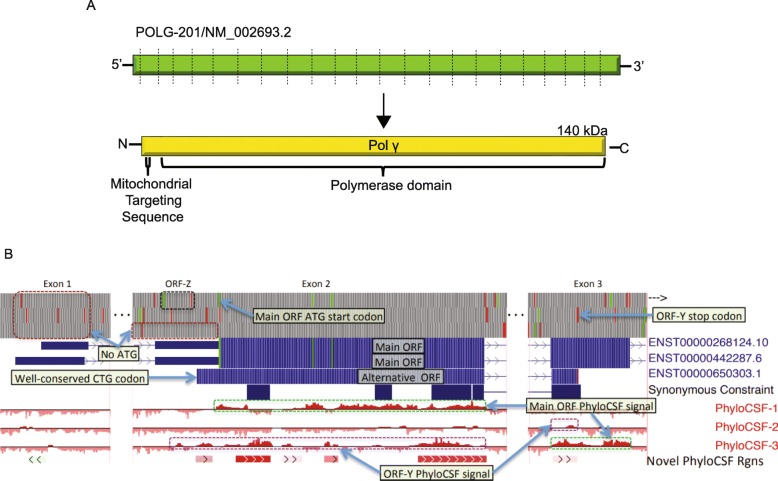
Architecture of the human *POLG* transcript. **a**. Diagram of the primary transcript for *POLG.* The dashed lines represent exon boundaries (not to scale). The protein product Pol γ contains a mitochondrial targeting sequence at the N-terminus and the rest of the protein consists of several domains that make up the DNA polymerase super-domain. **b**. UCSC Genome Browser [4] image of (from top to bottom) ATG codons (green) and stop codons (red) in the three theoretical reading frames on the minus strand of chromosome 15; first three exons of previously-annotated *POLG* transcripts ENST00000268124.10 and ENST00000442287.6; incomplete novel transcript ENST00000650303.1;Synonymous Constraint track showing regions with enhanced synonymous conservation; PhyloCSF tracks for the three minus strand frames; and PhyloCSF Candidate Coding Region (PCCR) track. The cluster of PCCRs suggests coding in some previously unannotated frame. The PhyloCSF signals suggest translation in chromosomal frame 3 in exon 2 and frame 2 in exon 3 (purple rectangles), terminating at a well-conserved stop codon in exon 3. There are no ATG codons in this frame in the 5′ portion of exon 2 or in any frame in exon 1 (dark red rectangles), suggesting that the initiation codon is not ATG. The coding region of ENST00000650303, ORF-Y, begins at a well-conserved CTG codon. The ATG and stop codon of a likely regulatory ORF, ORF-Z are also indicated (black rectangle)

*POLG* on the q arm of chromosome 15 encodes Pol γ, a 140 kDa catalytic subunit. The primary transcript (POLG-201 or NM_002693.2) for *POLG* is composed of 23 exons (Fig. 1a). The canonical AUG start codon is in exon 2 and the coding region continues into exon 23 [5]. Mutations in *POLG* are associated with mitochondrial disorders and represent the plurality of single gene causes of mitochondrial disorders [6]. Disorders related to *POLG* include mitochondrial epilepsy, autosomal recessive progressive external ophthalmoplegia, ataxia and many more. The age of onset for *POLG* related disorders can range anywhere from infancy to late adulthood [7]. Mutations have been mapped across the entire coding region of *POLG* from exons 2 to 23 (https://tools.niehs.nih.gov/polg/). The underlying mechanism for the progression of these diseases is typically related to a depletion of mtDNA or mutation of mtDNA due to a defective Pol γ [8]. There is currently a dearth of therapies for disorders caused by *POLG* mutations despite how widely it influences the population [7].

In the scanning model of translation, the 43S preinitiation ribosomal complex scans an mRNA until it encounters an AUG codon in a favorable initiation context [9]. Translation initiation occurs when the pre-bound initiator Met-tRNA binds to the initiation codon in the P-site of the ribosome [10, 11]. The transition from initiation to elongation is, in part, mediated by eIF5B dissociation [12]. For eukaryotes, the efficiency of initiation is dependent on the surrounding nucleotide context. The optimal sequence for translation initiation in mammals is known as the Kozak consensus [13]The optimal Kozak consensus in mammals and is GCCRCC***AUG***G (R = A or G), where the underlined nucleotides are the most important [13]. An ‘A’ at position − 3 is preferred over ‘G’, and a purine in that position is more important than a ‘G’ at the + 4 position (with respect to the ‘A’ in ***AUG***) [14].

Translation initiation can sometimes also occur at non-AUG codons with varying efficiency [15–20]. In mammals, CUG is widely regarded as the most efficient non-AUG codon [16]. In addition to the presence of a favorable initiation context, a stable RNA secondary structure beginning ~ 15 nt downstream of the initiation site increases initiation efficiency at non-AUG codons [21]. Such RNA structures are thought to pause the scanning 43S pre-initiation complex in the vicinity of the potential initiation codon and thus increase the propensity for initiation to occur [21].

In mammals, there are a handful of reported cases of functionally important non-AUG initiation codon utilization [20, 22]. In most cases, the alternative initiation site is utilized to produce a longer isoform than that produced from a downstream canonical AUG initiation site, with the latter being accessed via a process known as ‘leaky scanning’ [23]. In this process, a proportion of pre-initiation scanning 43S ribosomal complexes are able to scan past non-AUG or poor-context AUG initiation sites to initiate translation at downstream sites. Ribosome profiling studies have revealed potential widespread initiation at non-AUG codons [24, 25]. However, the biological relevance of many of these sites is not currently known. Further, addition of initiation inhibitors – such as lactimidomycin or harringtonine – that are used in many ribosome profiling studies, may artificially increase initiation at sites upstream of canonical initiation sites [26, 27]. It is thus necessary to combine ribosome profiling with orthogonal approaches such as comparative genomics and mass spectrometry.

Translation of very short open reading frames (ORFs that are shorter than ~ 30 codons) causes only a partial dissociation of post-termination ribosomes: the 60S subunit and deacylated tRNA are released conventionally but the 40S subunit can remain attached to the mRNA and resume scanning downstream [11, 28]. This can allow for an additional layer of translational control of other upstream open reading frames (uORFs) and/or the main ORF [25, 29].

Comparative analysis suggested a possible coding sequence overlapping POLG in an alternative reading frame, but with unidentified initiation codon. Our goals in this study were to seek ribosome profiling and mass spectrometry evidence that could confirm that the alternative coding sequence is translated, to determine its initiation codon, and to investigate the possible clinical significance of the novel coding sequence.

## Results

### PhyloCSF identification of two novel ORFs in the *POLG* mRNA

We initially found evidence of alternate-frame translation in *POLG* as part of a project to identify novel coding regions using PhyloCSF [30]. We had previously developed PhyloCSF [31] (Phylogenetic Codon Substitution Frequencies) to determine whether a given nucleotide sequence is likely to represent a functional, conserved protein-coding sequence by determining the likelihood ratio of its multi-species alignment under coding and non-coding models of evolution that use precomputed substitution frequencies for every possible pair of codons, trained on whole-genome data. To find novel coding regions we had computed PhyloCSF scores for every codon in the human genome in each of six reading frames, used a hidden Markov model to find potential coding intervals, and screened out intervals overlapping known coding or pseudogenic regions in the same frame or the antisense frame, leaving us with approximately 70,000 PhyloCSF Candidate Coding Regions (PCCRs), which were then prioritized by a machine learning algorithm and the first 1000 examined by expert manual annotators.

We found that a cluster of PCCRs on the minus strand of chromosome 15 are within exons 2 and 3 of *POLG* (Fig. 1b). Since we had previously screened out intervals overlapping known coding regions in the same frame, this indicated possible translation in an alternative reading frame. An alignment of 58 placental mammal genomes in the frame indicated by the PhyloCSF signal (the − 1 frame relative to the main ORF) indicated a partial ORF roughly coinciding with the signal and ending in a well-conserved stop codon (Supplementary Figure 1**)** but left ambiguous where the ORF started. There are no AUG codons in this reading frame 5′ of the PhyloCSF signal in exon 2, or in any frame in exon 1, suggesting that the ORF is initiated at a non-AUG start codon. The CUG codon with hg38 coordinates chr15:89333807–89,333,809 is conserved in all the aligned genomes and roughly coincides with the start of the PhyloCSF signal, so we investigated it further as a plausible candidate start codon. With this start, the candidate ORF, which we refer to as ORF-Y, would create a 260-amino acid protein with a PhyloCSF score of 412.1, which is significantly higher than could be expected to arise from a non-coding region of that length (*p* < 1 × 10^− 7^). We have included this translation in the GENCODE / Ensembl gene set as model ENST00000650303.1. Analysis of the sequence upstream of the CUG putative initiation codon revealed a second potential uORF, herein coined as ORF-Z (Supplementary Figure 2**)**.

### The overlapping portion of ORF-Y with the main CDS has a significantly reduced rate of synonymous substitutions in most mammals

**Fig. 2:**
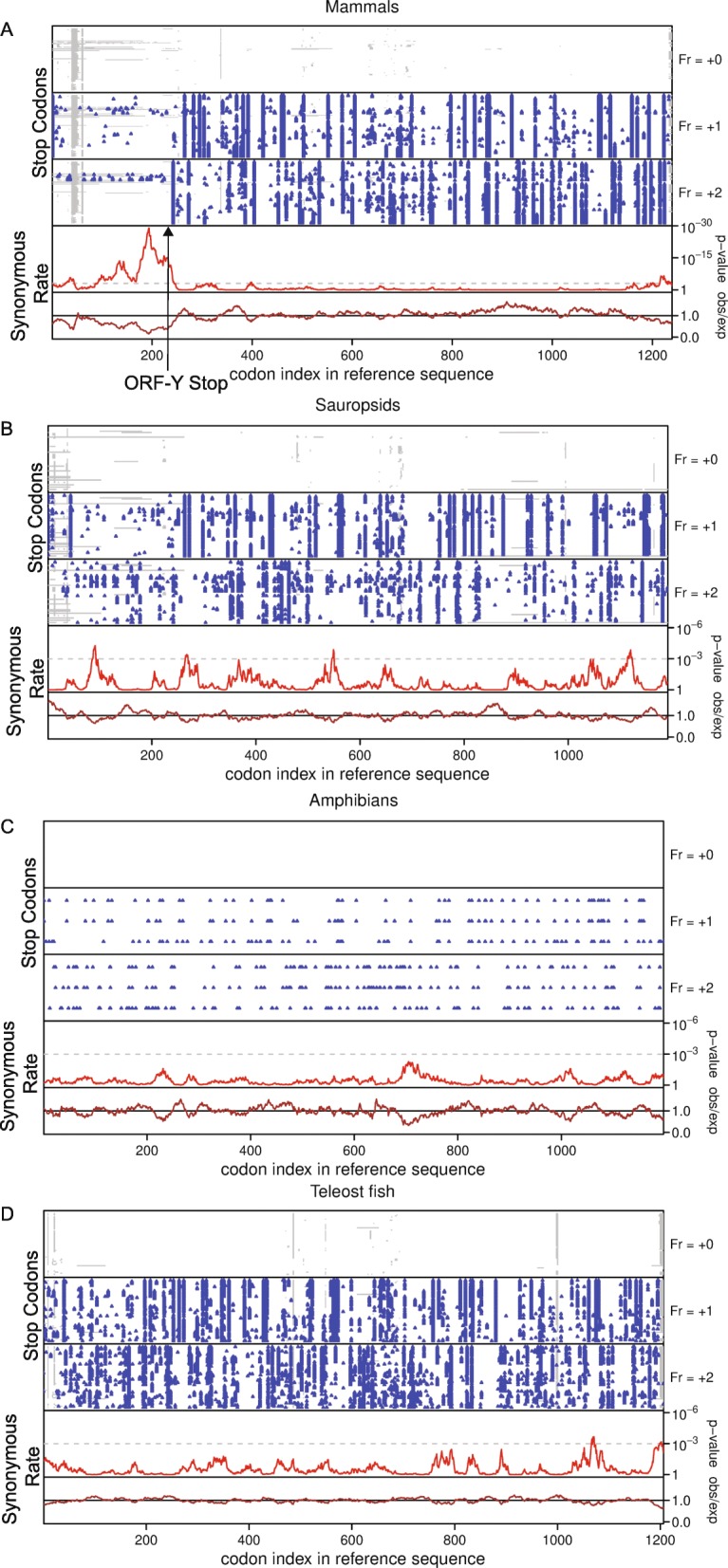
Synonymous site conservation in the POLG coding region for the major vertebrate clades. Clades shown are **a**. mammals, **b**. amphibians, **c**. sauropsids, and **d**. teleost fish. In each subfigure, the top panel shows the position of 0-frame stop codons in each sequence in the alignment. The following panels show the positions of stop codons in the + 1 and + 2 frames. The blue dots represent stop codons and the grey regions represent alignment gaps. The bottom two panels show the synonymous site conservation analysis, with the brown line showing the ratio of the observed number of synonymous substitutions within a given window to the number expected under a null model of neutral evolution at synonymous sites, and the red line showing the corresponding *p*-value. The horizontal grey dashed line indicates a *p* = 0.05 threshold after an approximate correction for multiple testing (namely scaling by [sliding window size]/[POLG ORF length]). All subfigures use a 25-codon sliding window. The stop codon of ORF-Y in mammals is indicated with a black arrow

Since translation in more than one frame can suppress synonymous substitutions, we assessed synonymous site conservation within the POLG ORF using the Synplot2 program [32]. Plots of stop codon positions in each of the three forward reading frames of the alignment were also generated (Fig. 2). In the mammalian alignment, a highly significant increase in synonymous site conservation was observed in the ORF-Y overlap region (783 nucleotides in *Homo sapiens*) (Fig. 2a). Enhanced synonymous site conservation in the POLG ORF disappears immediately after the ORF-Y stop codon. The presence of such a long, conserved stop codon free region argues against an RNA structural element being responsible for the synonymous site conservation.

A closer look at organisms in the mammalian clade revealed that all *POLG* sequences contain a conserved CUG codon in ORF-Y that is in a good initiation context, except for *Camelus ferus* (camel)*,* and three marsupial species: *Vombatus ursinus* (wombat)*, Phascolarctos cinerus* (koala)*,* and *Monodelphis domestica* (opossum). A fourth marsupial species, *Sarcophilus harisii* (Tasmanian devil), has a CUG codon in the correct frame but the surrounding sequence is dissimilar to all other mammals. Furthermore, these five organisms have stop codons in the − 1 frame shortly after the main ORF AUG start codon (Fig. 2a).

The disruption of ORF-Y in marsupials suggests that it became a protein-coding ORF de novo in placental mammals. This is confirmed by a 100-vertebrates codon alignment of ORF-Y, which shows that the early portion of ORF-Y is frameshifted in marsupials and platypus (Supplementary Figure 3). Furthermore, looking at the alignment in the second and third blocks, we see that there are many in-frame stop codons in marsupials and most of the non-mammal vertebrates. Finally, the synonymous substitution constraint as seen in Synplot2 analysis (Fig. 2a) appears to be restricted to placental mammals.

### Ribosome profiling of *POLG* reveals that ORF-Y is actively translated

**Fig. 3:**
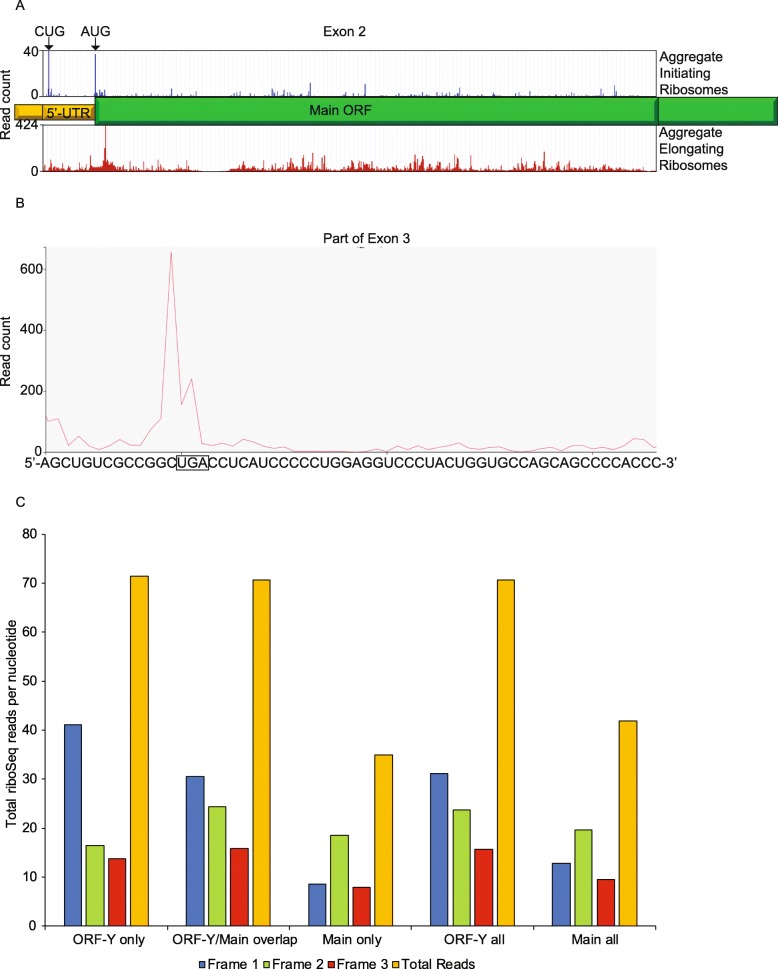
Ribosome profiling analysis of ORF-Y. Aggregated ribosome profiling data for all studies available on GWIPs-viz (subfigure **a**) and Trips-Viz (subfigures **b-c**). **a**. Ribosome profiling coverage of *POLG* exon 2. The top panel in blue shows the aggregate of initiating ribosome profiling experiments (samples treated with harringtonine or lactimidomycin) and the bottom panel in red shows the aggregate of elongating ribosome profiling experiments. **b**. Ribosome profiling coverage of part of exon 3 containing the ORF-Y ‘UGA’ stop codon (box). **c**. Read counts by frame for the regions covering ORF-Y only, the ORF-Y/Main ORF overlap, the Main ORF only, and then all of ORF-Y and the Main ORF

In order to verify translation of ORF_Y, we mined *H. sapiens* ribosome profiling data from an aggregate of studies using GWIPS-viz [33–35] and Trips-Viz [36]. Aggregate ribosome profiling reveals translation in the 5′-UTR at a comparable level to the beginning of the main ORF. Filtering ribo-seq data for samples treated with the initiation inhibitors lactimidomycin or harringtonine shows a comparable level of initiating ribosomes at the main ORF AUG start codon and at the upstream ORF-Y CUG codon (Fig. 3a). If ribosomes were translating both ORFs prior to the − 1 frame stop codon for ORF-Y, a step-wise decrease in ribosome density after this stop codon could be apparent. Looking at an aggregate of elongation ribosome profiling studies, reads were found to peak at the − 1 frame stop codon for ORF-Y (Fig. 3b). Looking at the framing of ribosomes, we see that in the region overlapping ORF-Y and the POLG ORF, the plurality of ribosomes are in frame 1 but in the nonoverlapping region of the POLG ORF, the plurality of ribosomes are in frame 2. Following this − 1 frame stop codon, the number of reads per nucleotide drops in half, further indicating that a fraction of ribosomes have already terminated at ORF-Y’s stop codon (Fig. 3c).

### The initiation context of ORF-Y is highly favorable despite using a non-ATG start codon

**Fig. 4:**
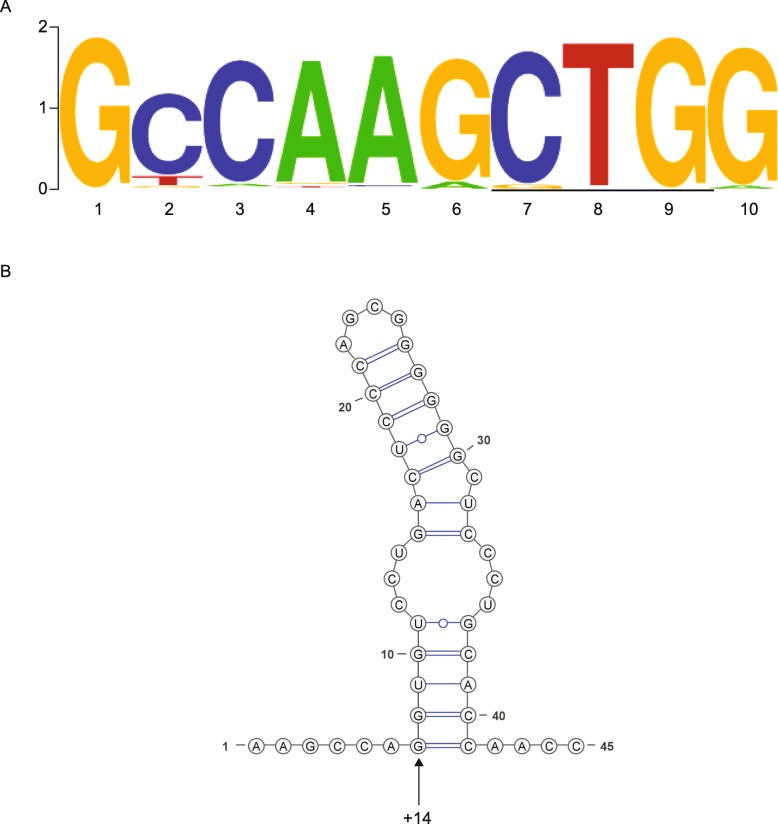
Initiation context of ORF-Y. **a**. Weblogo of initiation context sequences extracted from all mammalian *POLG* mRNA sequences that contain ORF-Y. The start codon is underlined. **b**. Representation of the consensus downstream RNA secondary structure for mammalian *POLG* mRNA sequences that contain ORF-Y. The structure was determined with RNAalifold. The arrow is pointing at the + 14 nucleotide, where the ‘G’ in ‘CUG’ is nucleotide 0

The CUG putative start codon has a strong initiation context (GCCAAG**CTG**G) that is highly conserved, though the initiator codon is GUG in a select few sequences (Fig. 4a). Specifically, the ‘G’ in the + 4 position and the ‘A’ in the − 3 position are the most favorable nucleotides for these critical positions.

To check for additional features that could provide a favorable context for initiation, the regions in 88 mammal genomes downstream of the CUG codon were aligned and probed for RNA secondary structure (Supplementary Figure 4, Fig. 4b). RNAalifold [37] predicted a stem loop with a bulge in the middle. Conservation of this stem-loop suggests that it may play a role in the promotion of initiation at the CUG codon. The stem-loop begins at the optimal distance (14 nt) from the initiation codon for pausing the 43S pre-initiation complex over the CUG codon [21].

### Proteomic evidence of active ORF-Y translation suggests that the peptide may harbor function

**Fig. 5:**
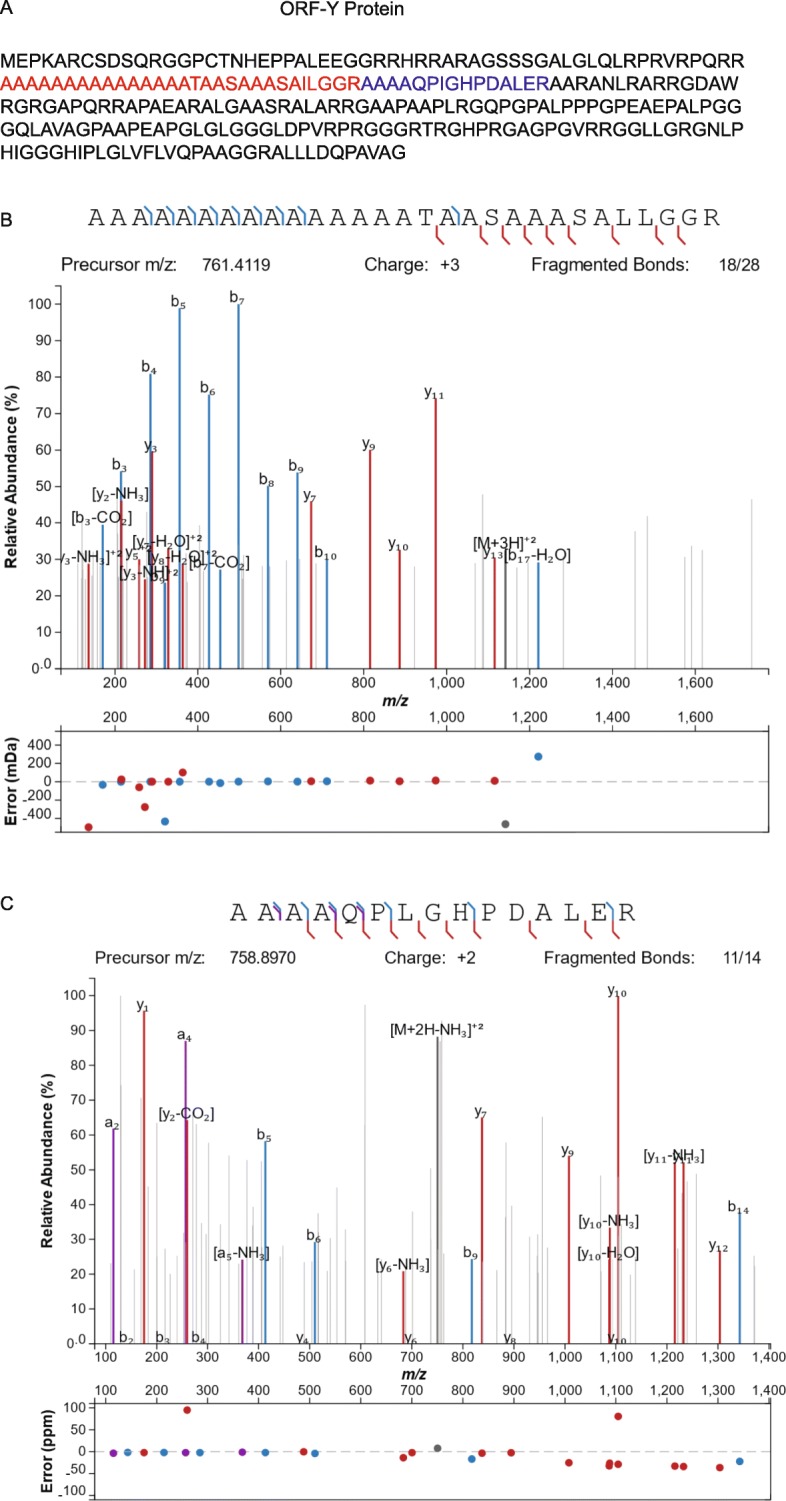
Mass spectrometry evidence for translation of ORF-Y. **a**. Predicted translation of human ORF-Y. The CUG initiation codon is presumed to translate to methionine. The two peptides detected by mass spectrometry are colored in blue and red. **b**. Spectra for the first (red) peptide. **c**. Spectra for the second (blue) peptide. The sequences of the fragmented ions and their abundances are shown in both **b** and **c**

We next investigated proteomic evidence for translation of ORF-Y, by reanalyzing the Kim et al., 2014 draft human proteome datasets [38] and searching against a set of candidate coding regions detected by PhyloCSF including the ORF-Y protein sequence [39]. Two unique peptides (AAAAQPJGHPDAJER and AAAAAAAAAAAAAAATAASAAASAJJGGR) were found only in CD8 T-cell samples mapping unambiguously to the candidate protein sequence (Fig. 5). This could suggest that the function of ORF-Y’s protein product is linked to an immune function, since high confidence peptides were not found in other cell types; however, mass spectrometry is not guaranteed to detect all expressed proteins, so it is possible that ORF-Y is expressed in other cell types as well. The first of these peptides confirms a previous identification made in the original Kim et al. analysis, and has since been confirmed in PeptideAtlas [40] across 7 additional experiments (PAp06322239). This further supports the translation of the proposed ORF-Y into a protein that is folded stably enough to be detected, suggesting it may have function. The protein product of ORF-Y for *H. sapiens* is predicted to have a transmembrane domain (TMHMM prediction software [41]). However, inspection of the ORF-Y protein products for representative members of other mammalian orders reveals that this predicted transmembrane domain is not conserved (Supplementary Figure 5A). An alanine repeat expansion appears to have occurred in some species, causing the TMHMM prediction software [41] to call some of these peptides as potential transmembrane domains (Supplementary Figure 6). Taking the portion of the ORF-Y peptide corresponding to the region of strongest POLG-frame synonymous site conservation (Fig. 2; region with *p* < 10^− 20^) and inputting it into the Eukaryotic Linear Motif (ELM) prediction server [42] yielded five potential functions (Supplementary Figure 5B). One of them, a predicted tankyrase binding motif, is plausible given that tankyrases are members of the poly ADP-ribose polymerase (PARP) family, DNA methylation and repair are some of the many functions of proteins in this family, and these functions are all related to the function of the POLG protein in DNA replication [43]. Two of the five predicted motifs are cleavage sites, and the other two are localization signals.

### ORF-Z is highly translated and probably regulatory

**Fig. 6:**
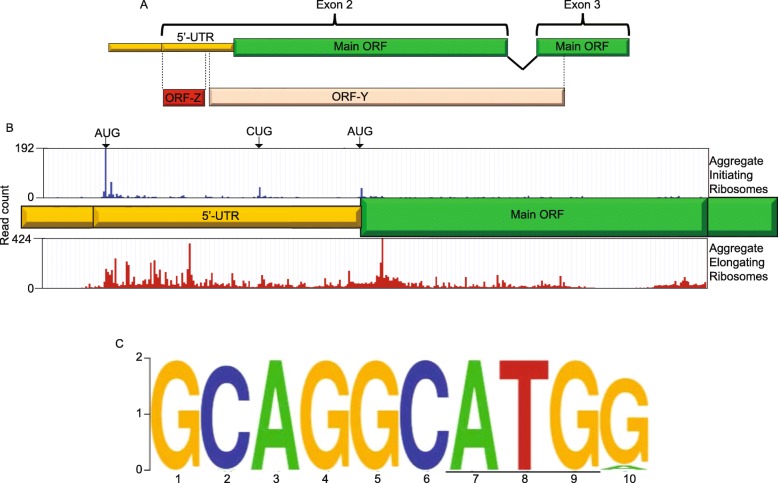
*POLG* contains a further upstream ORF-Z. **a**. Schematic of where ORF-Z is located relative to the architecture of *POLG*. **b**. Ribosome profiling data mined from GWIPs-viz. The top panel in blue represents initiating ribosomes while the bottom panel in red represents elongating ribosomes. Arrows indicate positions of the initiation codons of all three ORFs, which exactly match peaks in initiating ribosome coverage. **c**. Weblogo of ORF-Z initiation contexts extracted from mammalian *POLG* mRNA sequences that contain ORF-Y and at least 150 nucleotides of 5′ UTR. The start codon is underlined

Ribosome profiling indicates that translation initiation is potentially even more efficient at the AUG initiation codon of ORF-Z than at the CUG of ORF-Y or the main start codon (Fig. 6a and b, Fig. 1b**)**. The initiation context surrounding this upstream AUG is also favorable with a G at − 3 and a G at + 4 (Fig. 6c). The theoretical translation of ORF-Z is only 23 amino acids in length and not highly conserved, having a negative PhyloCSF score. However, CodAlignView [44] shows that the start and stop codons for ORF-Z and its reading frame are indeed well conserved across placental mammals (Supplementary Figure 2), suggesting that translation of ORF-Z, but not the encoded peptide, could be functionally important, for example by playing a regulatory role in translation of ORF-Y and/or the POLG ORF [45]. We also examined ORF-Z and ORF-Y ribosome profiling in both *Mus musculus and Rattus norvegicus* (Supplementary Figure 7)*.* We found that the ribosome footprints found in rats met the expected trend with a spike of reads at the ORF-Z and ORF-Y start codons. However, the footprints found in mouse are not what was expected. There is little translation in ORF-Y and there appears to be translation occurring 5′ of ORF-Z. This could be due to two different reasons. It could be possible that mice have loss the ability to translate ORF-Y. This could leave an open question of how, mechanistically, it could be behave differently in mouse and rat. Yet the Kozak context is the same in both species (Supplementary Figure 2) and the nucleotides involved in the downstream secondary structure are the same, with the exception of the fifth position of the first stem (a C in mice, and a U in rats) that does not affect the folding (in both species, the C or U base pair to a G, Supplementary Figure 4). Alternatively, it is possible that the set of ribosome profiling experiments in mice do not include the conditions needed for ORF-Y to be translated, especially since the diversity of ribosome profiling experiments available for humans is much larger than that of mice.

### Clinvar analysis reveals potentially harmful mutations in ORF-Y

Since mutations in *POLG* have been well documented in mitochondrial disease [7], we surveyed reported Clinvar variants within ORF-Z or ORF-Y that are synonymous or in the 5′-UTR with respect to the main ORF (Table 1). We found 41 Clinvar variants that do not to change the POLG amino acid sequence but that do affect the ORF-Y peptide, and one variant that changes an ORF-Z amino acid, though this one might not be as important since ORF-Z is likely a regulatory ORF rather than a coding one. Many of these mutations are listed as benign, perhaps owing to the fact that they appeared to be synonymous. Given the evidence that ORF-Y encodes a functional protein, such mutations should be re-evaluated for their possible clinical significance.

**Table 1:** Variants in ORF-Y and ORF-Z. Variants that are synonymous when translated in the reading frame of the main *POLG* ORF or that are listed as UTR variants, with their predicted effects on the translation product of ORF-Y or ORF-Z

rsID	Ref > Alt	Position (Anchor)	ORF-Y	ORF-Z
**768005050**	G>A	chr15:89333152 (GRCh38.p12)	no change	not in ORF
**1057522857**	C>T	chr15:89330213 (GRCh38.p12)	G259S	not in ORF
**750915606**	G>A	chr15:89333227 (GRCh38.p12)	P194S	not in ORF
**766842881**	G>C	chr15:89333233 (GRCh38.p12)	L192V	not in ORF
**1028326668**	C>T	chr15:89333239 (GRCh38.p12)	G190R	not in ORF
**886044612**	C>T	chr15:89333254 (GRCh38.p12)	G185S	not in ORF
**1057520491**	G>A	chr15:89333266 (GRCh38.p12)	P181S	not in ORF
**375445567**	G>A/C	chr15:89333271 (GRCh38.p12)	A179G	not in ORF
**1567194008**	A>G	chr15:89333283 (GRCh38.p12)	V175A	not in ORF
**1567194019**	C>T	chr15:89333287 (GRCh38.p12)	A174T	not in ORF
**779981823**	C>T	chr15:89333302 (GRCh38.p12)	G169R	not in ORF
**761417163**	G>A	chr15:89333332 (GRCh38.p12)	P159S	not in ORF
**558958919**	C>A/G/T	chr15:89333371 (GRCh38.p12)	A146T/A146S/A146P	not in ORF
**1057524724**	C>A	chr15:89333374 (GRCh38.p12)	A145S	not in ORF
**1057521700**	G>C	chr15:89333419 (GRCh38.p12)	L130V	not in ORF
**56221189**	C>A	chr15:89333422 (GRCh38.p12)	A129S	not in ORF
**376266682**	G>A	chr15:89333425 (GRCh38.p12)	R128W	not in ORF
**144439703**	G>A/C	chr15:89333491 (GRCh38.p12)	R106G/R106W	not in ORF
**774537232**	G>A/C/T	chr15:89333518 (GRCh38.p12)	L97I/L97V/L97F	not in ORF
**1241802528**	A>G	chr15:89333535 (GRCh38.p12)	I91T	not in ORF
**751225754**	C>A/G/T	chr15:89333545 (GRCh38.p12)	A88T/A88P/A88S	not in ORF
**745310138**	T>C	chr15:89333569 (GRCh38.p12)	I80V	not in ORF
**1555454318**	T>C	chr15:89333575 (GRCh38.p12)	S78G	not in ORF
**372383277**	C>A	chr15:89333578 (GRCh38.p12)	A77S	not in ORF
**796052878**	C>T	chr15:89333593 (GRCh38.p12)	A72T	not in ORF
**587781118**	T>A/C	chr15:89333596 (GRCh38.p12)	T71A/T71S	not in ORF
**587781117**	C>G/T	chr15:89333599 (GRCh38.p12)	A70T/A70P	not in ORF
**1453538834**	C>T	chr15:89333602 (GRCh38.p12)	A69T	not in ORF
**766501874**	C>T	chr15:89333605 (GRCh38.p12)	A68T	not in ORF
**570989155**	C>T	chr15:89333626 (GRCh38.p12)	A61T	not in ORF
**794727268**	C>A	chr15:89333641 (GRCh38.p12)	A56S	not in ORF
**587781116**	G>A	chr15:89333668 (GRCh38.p12)	R47C	not in ORF
**944054671**	T>C/G	chr15:89333695 (GRCh38.p12)	S38R/S38G	not in ORF
**1378670216**	C>G	chr15:89333701 (GRCh38.p12)	G36R	not in ORF
**535213599**	G>A/C	chr15:89333716 (GRCh38.p12)	R31G/R31C	not in ORF
**1482684558**	G>A	chr15:89333722 (GRCh38.p12)	R29C	not in ORF
**1060504037**	G>A	chr15:89333725 (GRCh38.p12)	R28W	not in ORF
**1057523280**	C>T	chr15:89333734 (GRCh38.p12)	E25K	not in ORF
**892999189**	G>A/C	chr15:89333740 (GRCh38.p12)	L23V/L23L	not in ORF
**750010376**	C>A	chr15:89333775 (GRCh38.p12)	R11L	not in ORF
**1284152513**	A>C	chr15:89333782 (GRCh38.p12)	S9A	not in ORF
**1057521902**	G>A	chr15:89333802 (GRCh38.p12)	P2L	not in ORF
**553331485**	T>C	chr15:89333821 (GRCh38.p12)	not in ORF	no change
**3087378**	G>A	chr15:89333834 (GRCh38.p12)	not in ORF	S20F

## Discussion

Mutations in *POLG* have been well documented in causing a range of diseases. The six leading disorders caused by *POLG* mutations are Alpers-Huttenlocher syndrome, childhood myocerebrohepatopathy spectrum, myoclonic epilepsy myopathy sensory ataxia, ataxia neuropathy spectrum, autosome recessive progressive external ophthalmoplegia, and autosome dominant progressive external ophthalmoplegia. Given that *POLG* mutations are the most prevalent single gene cause of mitochondrial disease and there is a lack of any evidence-based therapies, understanding translation dynamics of its mRNA is important. In addition, mutations in *POLG* have been implicated in Parkinsonism related symptoms and potentially accelerated aging. Both single- and bi-allelic inheritance of mutations can cause disease [7]. While the majority of non-synonymous *POLG* disease-correlated mutations are in the polymerase domain, there is a substantial number of reported mutations in the region that overlaps with ORF-Y. Given that synonymous mutations are less likely to affect the pathogenesis of disease, they have not been extensively discussed in the literature. While the function of the protein generated by ORF-Y is unknown, it is clearly conserved and subject to purifying selection (Figs. 2 and 4). What is remarkable is that *POLG* has existed in vertebrates but an overlapping ORF-Y has only recently arisen in placental mammals and has a protein product that likely has function. It may be that the primary event in the creation of both ORF-Z and ORF-Y was a transposon insertion, as a ~ 300 bp region of sequence containing the entirety of ORF-Z and the initiation codon of ORF-Y has been ‘repeat masked’ (http://repeatmasker.org) as a Mammalian-wide Interspersed Repeat (MIR) in both the Ensembl [46] and UCSC genome browsers [47] (~chr15:89333758–89,333,941). MIRs are an ancient transposon class within the SINE family, and these elements underwent a massive expansion prior to the radiation of placental mammals [48]. It is known that MIRs can ‘exonise’, and potentially contribute new functionality to existing protein-coding genes [49]. However, we note that the *POLG* MIR prediction is low scoring, and it is not consistently recapitulated in other mammalian genomes.

Both POLG and ORF-Y are presumably translated from the same transcripts meaning that they are subject to the same promoter driven regulation, and thus it is plausible that they might play roles in related pathways. Based on the ELM prediction of possible association with tankyrases, one could potentially predict that the ORF-Y protein may play a role in the maintenance of the mitochondrial genome. Without experimental evidence however, these hypotheses of ORF-Y protein function are simply speculation. We hope that in the future, researchers will take note of synonymous mutations in the region of *POLG* that overlaps with ORF-Y to see if there are links between mutations in the ORF-Y protein and particular disease phenotypes.

All known complete human transcripts of POLG that include ORF-Y also include several splice junctions 3′ of the ORF-Y stop codon, and thus one might expect that translation of ORF-Y would trigger Nonsense Mediated Decay (NMD), a cellular quality control pathway that is generally thought to degrade an mRNA if any Exon Junction Complexes (EJCs) are not removed by the ribosome the first time the mRNA molecule is translated [50]. However, the presence of two distinct overlapping translated ORFs on the same mRNA molecule might allow it to escape NMD. The stop codon of the POLG ORF lies in the final exon, so if the ribosome translates the POLG ORF the first time it translates the mRNA molecule, it will remove all of the EJCs and the molecule will escape NMD. Subsequent translation of ORF-Y on that same mRNA molecule will not trigger NMD because the EJCs will have already been removed. This model of NMD avoidance should be kept in mind when considering possible models of *POLG* translation dynamics and when choosing a system for experimental investigation of ORF-Y, because the triggers for NMD are thought to be different in non-mammals [51].

**Fig. 7:**
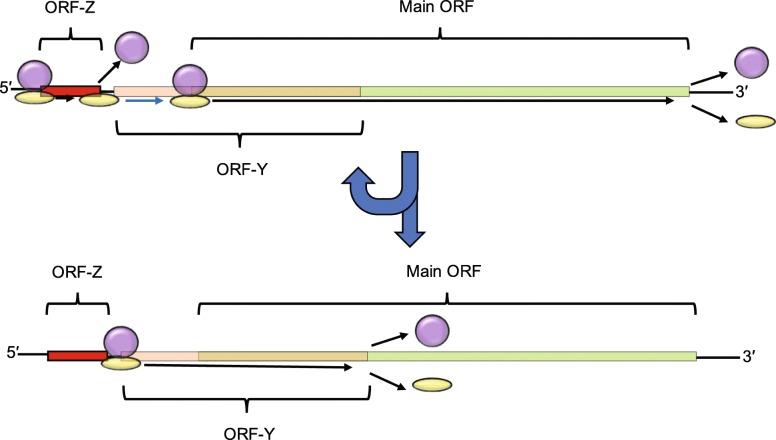
Model of translation. Schematic of how the translation of all three ORF’s is regulated. The yellow oval represents the small subunit and the large purple oval represents the large subunit. The red, pink, brown, and green portions of the mRNA correspond to ORF-Z, ORF-Y no overlap, ORF-Y/main CDS overlap, and main CDS only portions retrospectively. The thin black lines represent the UTR’s. The thin blue arrow represents the small subunit remaining attached to the mRNA after termination at the ORF-Z stop codon and reinitiating at the main CDS start codon

Given the distance between the stop codon of ORF-Z and the start codon of ORF-Y (Supplementary Figure 2), it is likely that ribosomal 40S subunits that remain associated with the mRNA after translation of the short ORF-Z may re-initiate at the POLG ORF rather than ORF-Y. This is because post-termination 40S subunits need to re-acquire initiation factors before they become initiation-competent, and the CUG of ORF-Y is positioned too close to the stop codon of ORF-Z to allow time for this to occur [11, 28]. Thus in the scanning model of initiation, the first ORF to be translated would often be ORF-Z followed by reinitiation at the POLG ORF thus, in the first round of translation, typically clearing EJCs and allowing for translation of ORF-Y in (some) subsequent rounds of translation without the risk of mRNA transcript degradation via NMD (Fig. 7). It is possible that ORF-Z plays a regulatory role controlling levels of ORF-Y and POLG ORF translation in response to changing cellular conditions.

## Conclusion

In this study, we have provided evidence for the translation of ORF-Y and for its initiation at a CUG codon in a favorable initiation context. There are only a handful of known dual-coding regions in the human genome that have such length and maintain both ORFs in different reading frames for the entire length of each ORF. These findings are interesting due to the clinical relevance of *POLG*. Phenotypes previously ascribed to POLG mutations may, in some cases, actually derive from changes in the ORF-Y product. Lastly, the existence of ORF-Z adds a new layer to the potential translational regulation of both the POLG ORF and ORF-Y.

## Methods

### Obtaining orthologous *POLG* sequences

To identify orthologs of *POLG* in different vertebrate clades, tblastn searches using selected reference species (mammals: *Homo sapiens* (NM_002693.2), sauropsids: *Gallus gallus* (XM_015292047.2), amphibians: *Xenopus tropicalis* (XM_002932235.4), teleost fish: *Danio rerio* (XM_001921095.6)) were performed. Default parameters were used except the number of top hits was expanded to 500, the database used was the RefSeq RNA database, and the organism parameter was limited to the respective vertebrate clade. To reduce detection of sequences that are not orthologous, a minimum query cover threshold of 80% was set. Hits that had ‘partial mRNA’ in the name were removed. Sequences were retrieved from NCBI. When multiple transcript isoforms were present for a given species, the sequence with the highest bit score was chosen.

### Synonymous substitution rate analysis

The POLG ORF sequences for each clade were translated and aligned with MUSCLE [52] and the amino acid alignments were used to generate codon-based nucleotide alignments with EMBOSS tranalign [53]. Synonymous site conservation was assessed using Synplot2 [32]. Alignments were mapped to the reference species in each clade by removing all alignment columns that contained an alignment gap in the reference sequence. For the mammalian clade analysis, sequences from *Bison bison bison* (XM_010841133.1), *Oryctolagus cuniculus* (XM_017337563), and *Camelus ferus* (XM_006192570) were removed due to poor alignment (these are predicted, not experimentally verified, transcripts and it is likely that they are misannotated). Similarly, for the teleost fish analysis, the *Austrafundulus limnaeus* (XM_014005514) sequence was removed due to poor alignment.

#### PhyloCSF, CodAlignView, and synonymous constraint track

PhyloCSF scores for ORF-Y and ORF-Z were computed using the 58mammals parameter set and the default mle and AsIs options, applied to the complete ORF excluding the final stop codon. The *p*-value for the PhyloCSF score for ORF-Y was calculated using the non-coding model of PhyloCSF-Ψ described by Lin et al. [31] with coefficients μ_N_ = − 18.6390680431, A_N_ = 17.5118631166, BN = 0.728619879775. Alignments used as input to PhyloCSF and shown in CodAlignView were extracted from the 58 placental-mammal subset of the 100-vertebrates hg38 alignments, downloaded from the UCSC Genome Browser [4]. The Synonymous Constraint track shown in the browser image of Fig. 1b used the Synonymous Constraint track hub, available at https://data.broadinstitute.org/compbio1/SynonymousConstraintTracks/trackHub/hub.txt.

#### Ribosome profiling analysis

The GWIPS-viz [33–35] and Trips-Viz [36] databases were mined for ribosome profiling data on May 27th, 2019 and May 28th, 2019 respectively. For GWIPS-viz, default parameters were used with the exception that data from initiating ribosomes (P-site) was included as well. All studies available at the time were included in the analysis. We mined Trips-Viz for ribosome profiling data for *M. musculus* and *R. norvegicus* on XXX …

#### 5′-UTR alignment and initiation context motif generation

For the mammalian clade, we selected sequences that include an annotated 5′-UTR of length at least 100 nucleotides (ORF-Y analysis) or 150 nucleotides (ORF-Z analysis). From this subset, the entire annotated 5′-UTR region was aligned with MUSCLE [54] at a nucleotide level and visualized with SeaView [55]. The ORF-Y and ORF-Z initiation contexts were extracted from the alignment and sequence logos generated using the Berkeley Web Logo website (https://weblogo.berkeley.edu/logo.cgi).

#### Phylogenetic RNA secondary structure conservation

Sequences in the mammalian clade that contain a conserved ORF-Y CUG putative initiation codon were used for this analysis (this included all mammalian sequences except those from *Camelus ferus*: XM_006192570, *Vombatus ursinus*: XM_027851422, *Phascolarctos cinereus*: XM_020964921, *Monodelphis domestica*: XM_007479352, and *Sarcophilus harrisii*: XM_003755551). The portion of RNA that was aligned with MUSCLE [54] consisted of the sequence begining eight nucleotides 3′ of the ‘C’ of the CUG initiation codon and up to the POLG start codon. This sequence alignment was folded on the RNAalifold [37] server (http://rna.tbi.univie.ac.at/cgi-bin/RNAWebSuite/RNAalifold.cgi). The consensus sequence and fold were visualized using the Visualization Applet for RNA secondary structure software (VARNA).

#### Identification of peptides mapping to ORF-Y

The raw data published by Kim et al. [38] covering 30 tissues in 85 HCD (higher-energy collisional dissociation) mass spectrometry experiments was downloaded from the PRIDE database [56] (PXD000561, PXD002967) and converted to mzML format. These mzML spectra were searched using multiple search engines in a high confidence OpenMS [57] workflow as described by Wright et al. [39] and Weisser et al. [58] The spectra were search against a sequence database composed of all GENCODE v27 protein coding transcripts and PhyloCSF Candidate Coding Regions [29]; an equally sized decoy database generated using DecoyPYrat [59] was concatenated and used to control FDR. Peptides were filtered to a posterior error probability of less than 0.01 and required to be significant in multiple search engines; a minimum and maximum length of 6 and 30 amino acids respectively was set; a maximum of 2 missed cleavages were allowed, and peptides containing certain modifications, such as deamidation were excluded. The two ORF-Y peptides AAAAQPJGHPDAJER and AAAAAAAAAAAAAAATAASAAASAJJGGR were identified in the Adult CD8 T Cell experiments with a spectral posterior error probability of 0.00024 and 0.00138 respectively. The spectra matching these peptides were then extracted for further manual inspection. The Peptide Atlas link to the other proteomic experiments identifying the peptide AAAAQPJGHPDAJER is https://db.systemsbiology.net/sbeams/cgi/PeptideAtlas/GetPeptide?atlas_build_id=479&searchWithinThis=Peptide+Name&searchForThis=PAp06322239&action=QUERY.

### Clinvar analysis

On the NCBI variation viewer (https://www.ncbi.nlm.nih.gov/variation/view/), transcript variant 1 for *POLG* (NM_002693.2) was used as a query. Variants were then filtered to be single nucleotide variants, clinvar variants, and synonymous or 5′-UTR variants. All the variants found in exons 2 or 3 that matched these criteria were downloaded. Variants that were not within ORF-Y or ORF-Z were discarded. The remaining variants were mapped to ORF-Y or ORF-Z and the effect on the protein product was predicted. There were no clinvar indels for this region found.

## Supplementary information

**Additional file 1: Figure S1** CodAlignView of ORF-Y. Alignment of ORF-Y sequences from 58 placental mammals, color coded using CodAlignView (https://data.broadinstitute.org/compbio1/cav.php) (see legend). Insertions relative to the human sequence are not shown. The black outlined box indicates the ATG start codon of the POLG ORF. Orangutan, baboon, panda, chinese hamster, and Tibetan antelope sequences were excluded because they include frame-shifting indels in the (essential) POLG ORF which suggests they contain sequence or alignment errors. The ORF-Y initial CTG codon, TGA stop codon, and reading frame are conserved in all aligned species, except for an early stop codon in sheep.

**Additional file 2: Figure S2** CodAlignView of ORF-Z: Alignment in 58 placental mammals of ORF-Z and 29 downstream codons (gray). Black boxes indicate the start codons of ORF-Y (out-of-frame CTG) and POLG (in-frame ATG). The start codon, stop codon, and open reading frame of ORF-Z are conserved in all species except orangutan and megabat, suggesting that there has been selection to preserve the open reading frame. On the other hand, substitutions within ORF-Z are predominantly non-synonymous (red and dark green), suggesting a lack of purifying selection on the amino acid sequence. Consequently, we hypothesize that this is a regulatory uORF.

**Additional file 3: Figure S3** Vertebrate CodAlignView of ORF-Y. Alignment of ORF-Y sequences from 100 vertebrates, color coded using CodAlignView (see legend from Supplementary Figure 1). Insertions relative to the human sequence are not shown. The presence of frame-shifting indels and in-frame stop codons show that ORF-Y is not conserved beyond placental mammals.

**Additional file 4: Figure S4** Alignment showing conserved RNA secondary structure. The mammal alignment of the sequence from five nucleotides downstream of the CUG putative start codon up to the POLG start codon that was used by RNAalifold to predict a conserved RNA structure. Compensatory mutations are boxed and shaded with light blue.

**Additional file 5 **: **Figure S5**: Potential functional regions of the ORF-Y protein. a. Predicted ORF-Y protein sequences from representatives (*Homo sapiens*, *Mus musculus*, *Orcinus orca*, and *Myotis lucifugus*) of different mammalian orders were submitted to the TMHMM server for transmembrane domain prediction. Each vertical red bar represents the likelihood of a position being contained within a transmembrane domain; the blue line indicates whether the portion of the protein is predicted to be intracellular; and the purple line indicates whether the portion of the protein is predicted to be extracellular. The color of the horizontal line near the top of each plot indicates, for each position, whether it is most likely to be intracellular, transmembrane, or extracellular. b. Possible motifs predicted by the ELM database for the portion of the ORF-Y protein that is most conserved.

**Additional file 6: Figure S6** Alignment of ORF-Y protein sequences: The sequences from the same organisms in Supplementary Figure 3 from the ORF-Y sequence were translated and aligned with MUSCLE [46]. A black box is around the poly-alanine expansion found primarily in primates that appears as a predicted transmembrane domain in prediction software.

**Additional file 7: Figure S7** Ribosome profiling data from both *Mus musculus* and *Rattus norvegicus* mined from Trips-Viz. The red arrow and box indicate the location of the AUG for ORF-Z and the yellow arrow and box indicate the location of the CUG for ORF-Y.

## Data Availability

All software is publicly available and readily available at a GitHub page created for this article (https://github.com/YousufAKhan/POLG_Khan_Jungreis_et_al). Refer to the materials and methods for specific links to each dataset for each method. Accession numbers can be found in the materials and methods for relevant sections.

## Abbreviations

ETC: Electron transport chain
kDa: Kilodalton
mtDNA: Mitochondrial DNA
MUSCLE: MUltiple Sequence Comparison by Log-Expectation
ORF: Open reading frame
OXPHOS: Oxidative-phosphorylation
PhyloCSF: Phylogenetic Codon Substitution Frequencies
*POLG*: DNA polymerase subunit gamma, catalytic subunit
Ribo-seq: Ribosome profiling
rRNA: Ribosomal RNA
tRNA: Transfer RNA
UTR: Untranslated region
