# Supplementary material for: Evidence for a novel overlapping coding sequence in POLG initiated at a CUG start codon
Source: BMC Genet. 2020 Mar 6;21:25. doi: 10.1186/s12863-020-0828-7 (PMC7059407; doi:10.1186/s12863-020-0828-7)
Supplement: Supplementary file 1 — Additional file 1: Figure S1 CodAlignView of ORF-Y. Alignment of ORF-Y sequences from 58 placental mammals, color coded using CodAlignView (https://data.broadinstitute.org/compbio1/cav.php) (see legend). Insertions relative to the human sequence are not shown. The black outlined box indicates the ATG start codon of the POLG ORF. Orangutan, baboon, panda, chinese hamster, and Tibetan antelope sequences were excluded because they include frame-shifting indels in the (essential) POLG ORF which suggests they contain sequence or alignment errors. The ORF-Y initial CTG codon, TGA stop codon, and reading frame are conserved in all aligned species, except for an early stop codon in sheep. [file 12863_2020_828_MOESM1_ESM.pdf]

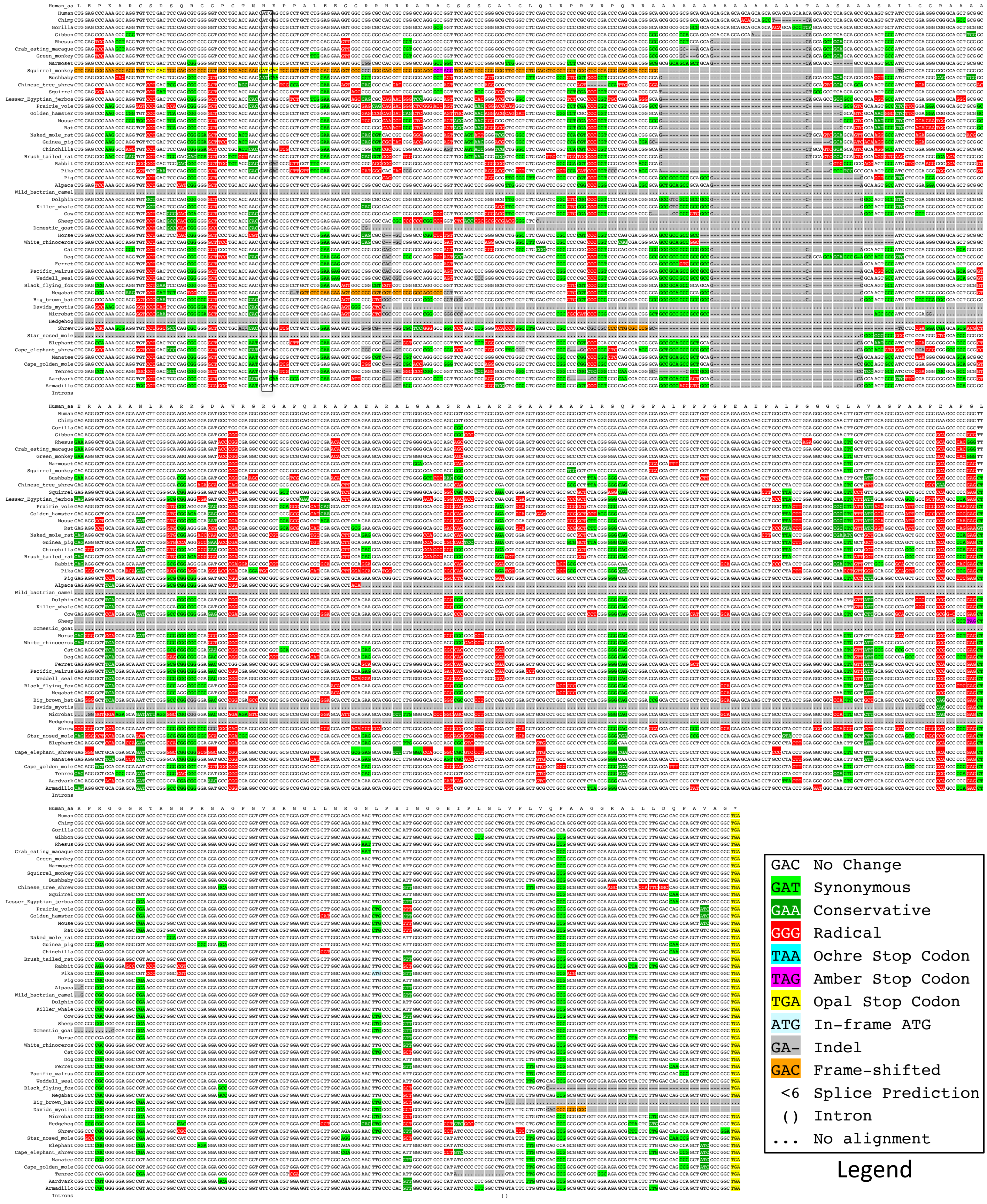
GAC No Change
GAT Synonymous
GAA Conservative
GGG Radical
TAA Ochre Stop Codon
TAG Amber Stop Codon
TGA Opal Stop Codon
ATG In-frame ATG
GA- Indel
GAC Frame-shifted
<6 Splice Prediction
() Intron
... No alignment
Legend
