# Supplementary material for: Evidence for a novel overlapping coding sequence in POLG initiated at a CUG start codon
Source: BMC Genet. 2020 Mar 6;21:25. doi: 10.1186/s12863-020-0828-7 (PMC7059407; doi:10.1186/s12863-020-0828-7)
Supplement: Supplementary file 2 — Additional file 2: Figure S2 CodAlignView of ORF-Z: Alignment in 58 placental mammals of ORF-Z and 29 downstream codons (gray). Black boxes indicate the start codons of ORF-Y (out-of-frame CTG) and POLG (in-frame ATG). The start codon, stop codon, and open reading frame of ORF-Z are conserved in all species except orangutan and megabat, suggesting that there has been selection to preserve the open reading frame. On the other hand, substitutions within ORF-Z are predominantly non-synonymous (red and dark green), suggesting a lack of purifying selection on the amino acid sequence. Consequently, we hypothesize that this is a regulatory uORF. [file 12863_2020_828_MOESM2_ESM.pdf]

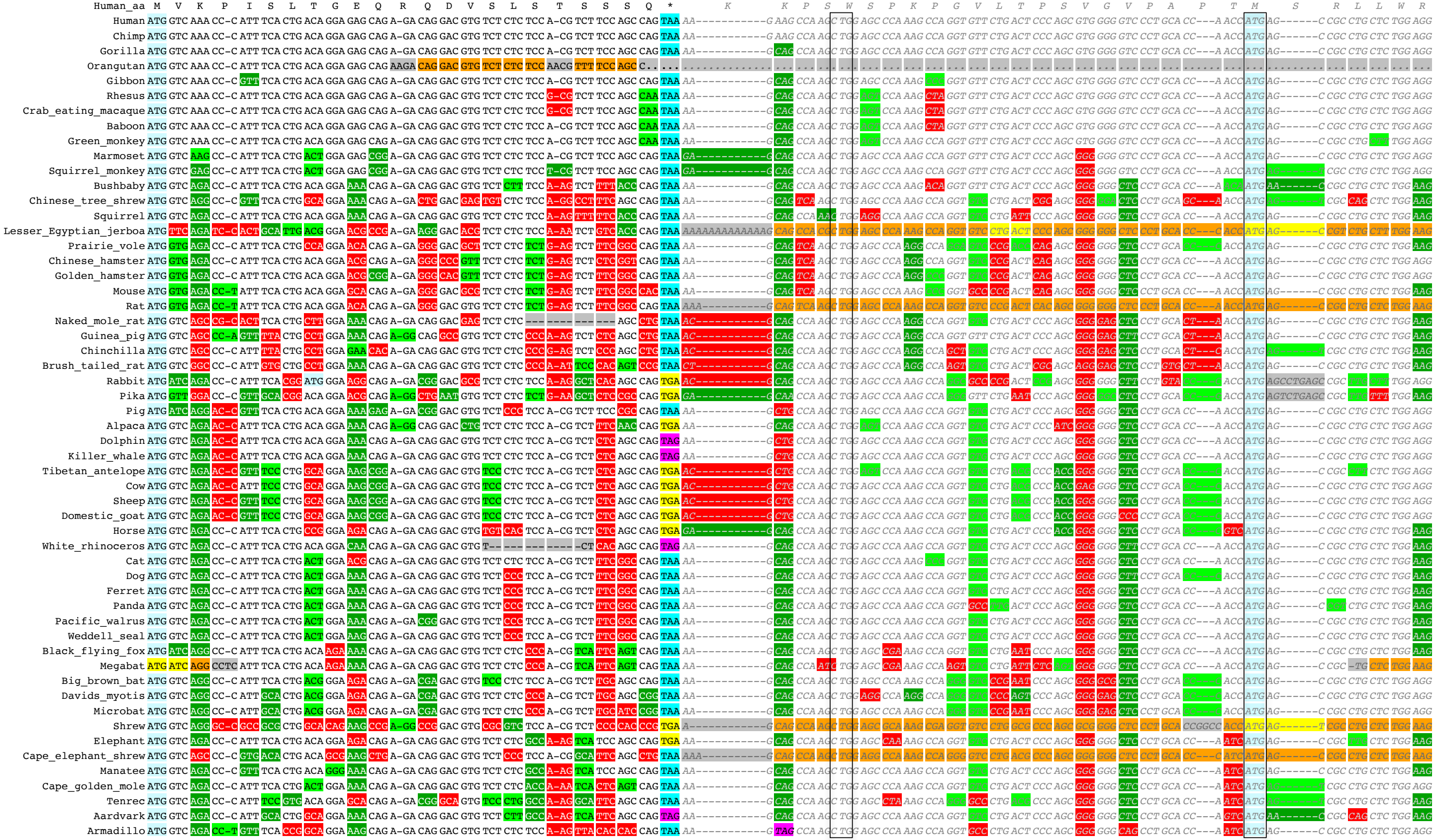
Human_aa M V K P I S L T G E Q R Q D V S L S T S S S Q *
Human
Chimp
Gorilla
Orangutan
Gibbon
Rhesus
Crab_eating_macaque
Baboon
Green_monkey
Marmoset
Squirrel_monkey
Bushbaby
Chinese_tree_shrew
Squirrel
Lesser_Egyptian_jerboa
Prairie_vole
Chinese_hamster
Golden_hamster
Mouse
Rat
Naked_mole_rat
Guinea_pig
Chinchilla
Brush_tailed_rat
Rabbit
Pika
Pig
Alpaca
Dolphin
Killer_whale
Tibetan_antelope
Cow
Sheep
Domestic_goat
Horse
White_rhinoceros
Cat
Dog
Ferret
Panda
Pacific_walrus
Weddell_seal
Black_flying_fox
Megabat
Big_brown_bat
Davids_myotis
Microbat
Shrew
Elephant
Cape_elephant_shrew
Manatee
Cape_golden_mole
Tenrec
Aardvark
Armadillo
