# Supplementary material for: Evidence for a novel overlapping coding sequence in POLG initiated at a CUG start codon
Source: BMC Genet. 2020 Mar 6;21:25. doi: 10.1186/s12863-020-0828-7 (PMC7059407; doi:10.1186/s12863-020-0828-7)

Extended Figure 4

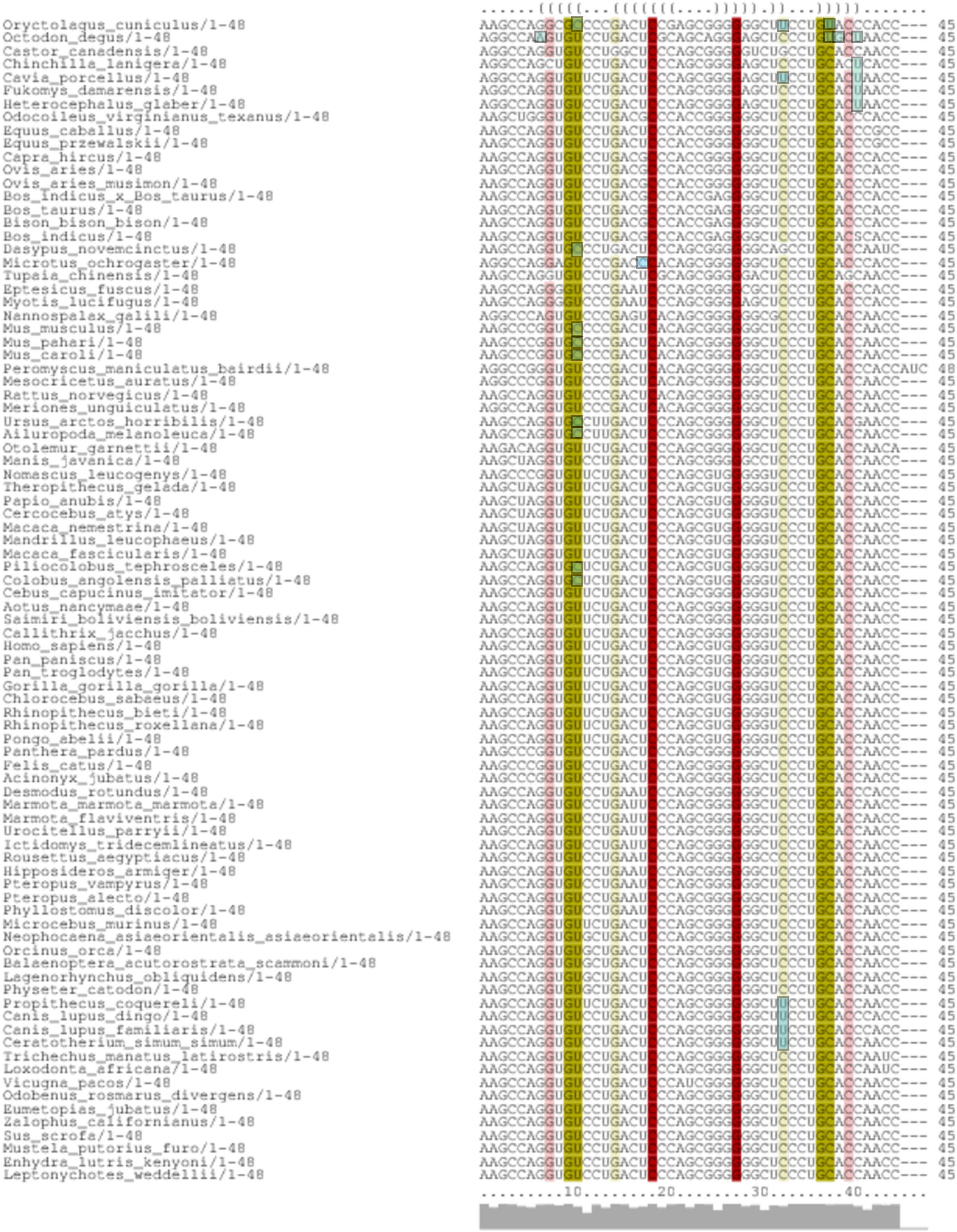

Supplement: Supplementary file 4 — Additional file 4: Figure S4 Alignment showing conserved RNA secondary structure. The mammal alignment of the sequence from five nucleotides downstream of the CUG putative start codon up to the POLG start codon that was used by RNAalifold to predict a conserved RNA structure. Compensatory mutations are boxed and shaded with light blue. [file 12863_2020_828_MOESM4_ESM.pdf]
