# Supplementary material for: Evidence for a novel overlapping coding sequence in POLG initiated at a CUG start codon
Source: BMC Genet. 2020 Mar 6;21:25. doi: 10.1186/s12863-020-0828-7 (PMC7059407; doi:10.1186/s12863-020-0828-7)
Supplement: Supplementary file 5 — Additional file 5 : Figure S5: Potential functional regions of the ORF-Y protein. a. Predicted ORF-Y protein sequences from representatives (Homo sapiens, Mus musculus, Orcinus orca, and Myotis lucifugus) of different mammalian orders were submitted to the TMHMM server for transmembrane domain prediction. Each vertical red bar represents the likelihood of a position being contained within a transmembrane domain; the blue line indicates whether the portion of the protein is predicted to be intracellular; and the purple line indicates whether the portion of the protein is predicted to be extracellular. The color of the horizontal line near the top of each plot indicates, for each position, whether it is most likely to be intracellular, transmembrane, or extracellular. b. Possible motifs predicted by the ELM database for the portion of the ORF-Y protein that is most conserved. [file 12863_2020_828_MOESM5_ESM.pdf]

Supplementary Figure 5

A

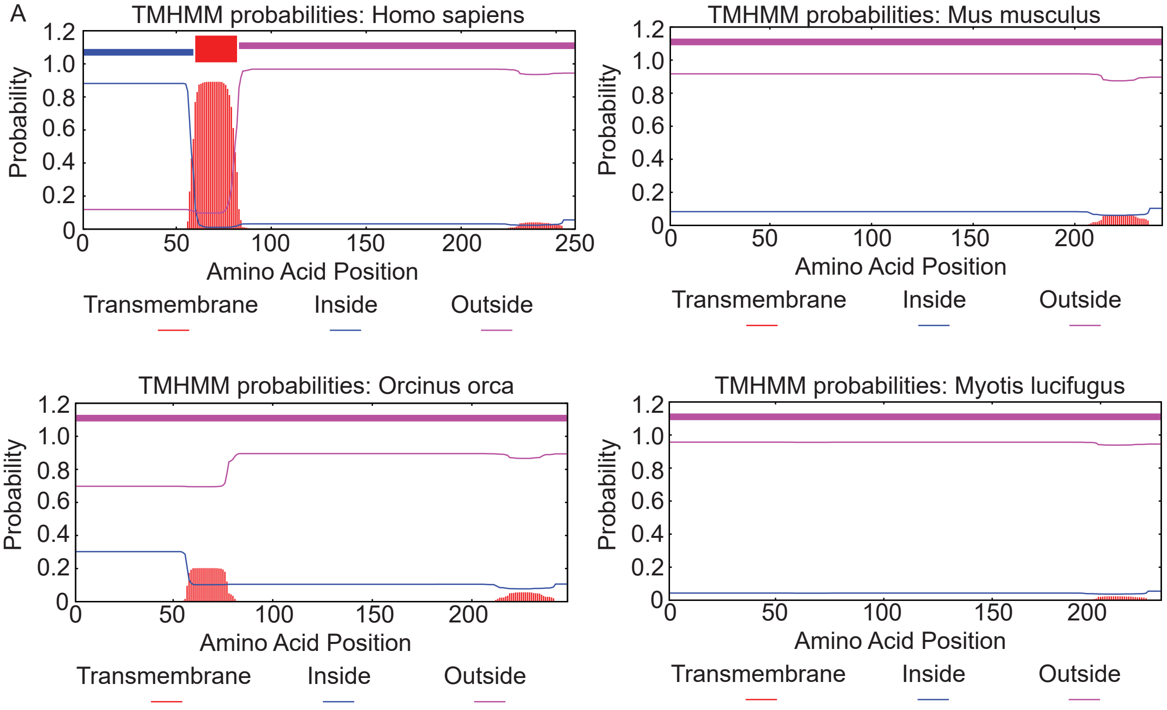

B

GHPRGAGPGVRRGGLL

ELM Name	Instances (Matched Sequence)	Positions	ELM Description	Cell Compartment	Probability
**CLV_NRD_NRD_1**	RRG	11-13	N-Arg dibasic convertase (NRD/Nardilysin) cleavage site (X-|-R-K or R-|-R-X).	extracellular, golgi apparatus, cell surface	7.465x10^{-3}
**CLV_PCSK_KEX2_1**	RRG	11-13	Yeast kexin 2 cleavage site (K-R-|-X or R-R-|-X).	extracellular, Golgi apparatus	7.973x10^{-3}
**DOC_ANK_TNKS_1**	PRGAGPGV	3-10	The Tankyrase binding motif interacts with the ankyrin repeat domain region in Tankyrase-1 and Tankyrase-2 to facilitate the PARsylation of the target proteins.	nucleus, cytosol, chromosome, telomeric region, spindle pole, exon-exon junction complex, beta-catenin destruction complex	3.538x10^{-4}
**TRG_ER_diArg_1**	VRR	10-12	The di-Arg ER retention motif is defined by two consecutive arginine residues (RR) or with a single residue insertion (RXR). The motif is completed by an adjacent hydrophobic/arginine residue which may be on either side of the Arg pair.	endoplasmic reticulum membrane, integral protein, ER-golgi transport vesicle membrane, endoplasmic reticulum membrane, golgi -ER transport vesicle membrane, rough endoplasmic reticulum, endoplasmic reticulum cisterna, cytosol	5.369x10^{-3}
**TRG_LysEnd_APsAcLL_1**	RRGGLL	11-16	Sorting and internalisation signal found in the cytoplasmic juxta-membrane region of type I transmembrane proteins. Targets them from the Trans Golgi Network to the lysosomal-endosomal-melanosomal compartments. Interacts with adaptor protein (AP) complexes	cytosol, endocytic vesicle	2.758x10^{-3}
