# Supplementary material for: Evidence for a novel overlapping coding sequence in POLG initiated at a CUG start codon
Source: BMC Genet. 2020 Mar 6;21:25. doi: 10.1186/s12863-020-0828-7 (PMC7059407; doi:10.1186/s12863-020-0828-7)
Supplement: Supplementary file 6 — Additional file 6: Figure S6 Alignment of ORF-Y protein sequences: The sequences from the same organisms in Supplementary Figure 3 from the ORF-Y sequence were translated and aligned with MUSCLE [46]. A black box is around the poly-alanine expansion found primarily in primates that appears as a predicted transmembrane domain in prediction software. [file 12863_2020_828_MOESM6_ESM.pdf]

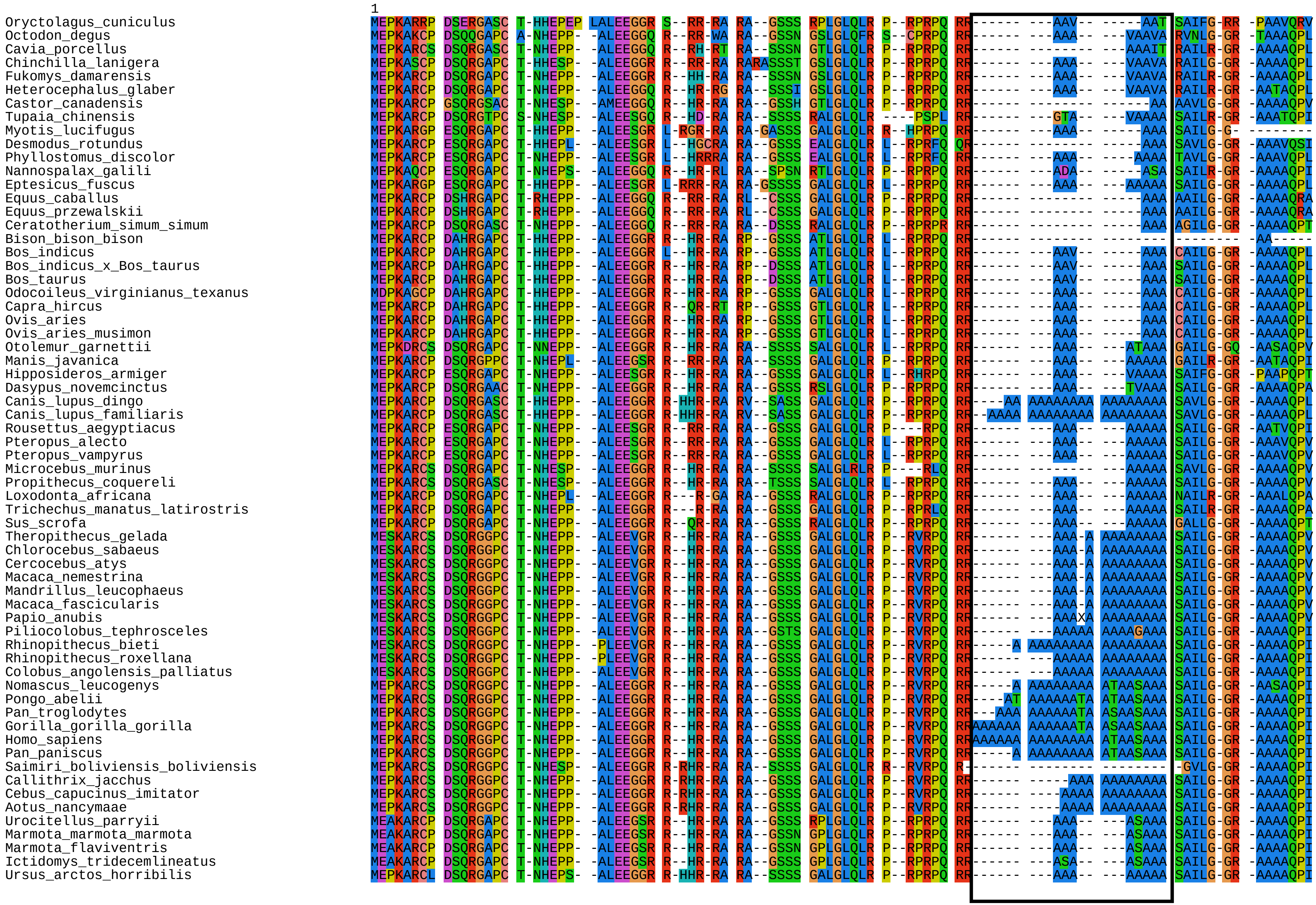
1
Oryctolagus_cuniculus
Octodon_degus
Cavia_porcellus
Chinchilla_lanigera
Fukomys_damarensis
Heterocephalus_glaber
Castor_canadensis
Tupaia_chinensis
Myotis_lucifugus
Desmodus_rotundus
Phyllostomus_discolor
Nannospalax_galili
Eptesicus_fuscus
Equus_caballus
Equus_przewalskii
Ceratotherium_simum_simum
Bison_bison_bison
Bos_indicus
Bos_indicus_x_Bos_taurus
Bos_taurus
Odocoileus_virginianus_texanus
Capra_hircus
Ovis_aries
Ovis_aries_musimon
Otolemur_garnettii
Manis_javanica
Hipposideros_armiger
Dasypus_novemcinctus
Canis_lupus_dingo
Canis_lupus_familiaris
Rousettus_aegyptiacus
Pteropus_alecto
Pteropus_vampyrus
Microcebus_murinus
Propithecus_coquereli
Loxodonta_africana
Trichechus_manatus_latirostris
Sus_scrofa
Theropithecus_gelada
Chlorocebus_sabaeus
Cercocebus_atys
Macaca_nemestrina
Mandrillus_leucophaeus
Macaca_fascicularis
Papio_anubis
Piliocolobus_tephrosceles
Rhinopithecus_bieti
Rhinopithecus_roxellana
Colobus_angolensis_palliatus
Nomascus_leucogenys
Pongo_abelii
Pan_troglodytes
Gorilla_gorilla_gorilla
Homo_sapiens
Pan_paniscus
Saimiri_boliviensis_boliviensis
Callithrix_jacchus
Cebus_capucinus_imitator
Aotus_nancymaae
Urocitellus_parryii
Marmota_marmota_marmota
Marmota_flaviventris
Ictidomys_tridecemlineatus
Ursus_arctos_horribilis

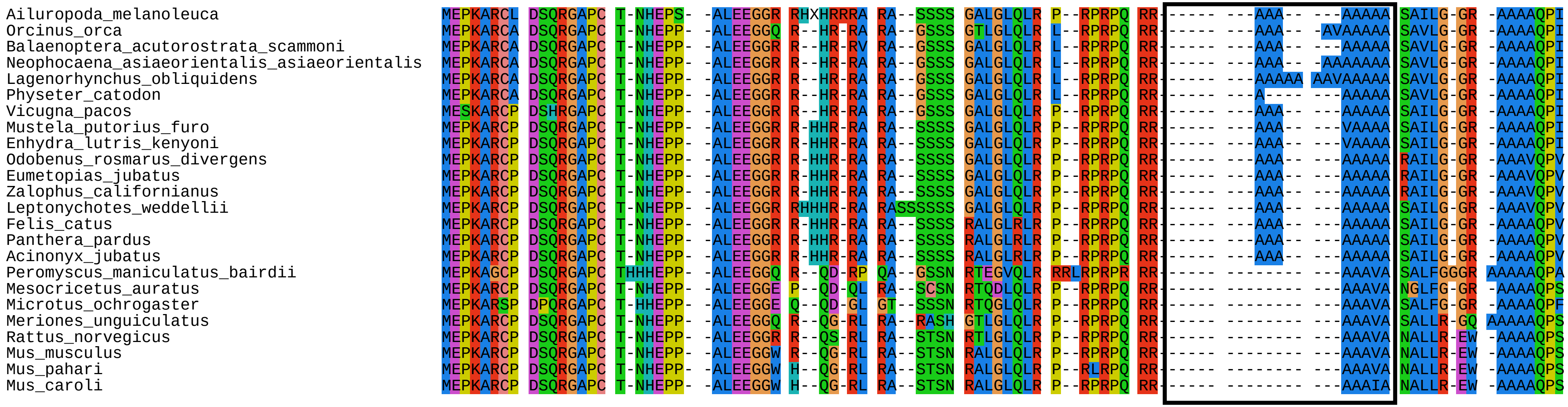
Ailuropoda_melanoleuca MEPKARCL DSQRGAPC T-NHEPS- -ALEEGGR RHXHRRRA RA--SSSS GALGLQLR P--RPRPQ RR------- ---AAA-- ---AAAAA SAILG-GR -AAAAQPI
Orcinus_orca MEPKARCA DSQRGAPC T-NHEPP- -ALEEGGQ R--HR-RA RA--GSSS GTLGLQLR L--RPRPQ RR------- ---AAA-- -AVAAAAA SAVLG-GR -AAAAQPI
Balaenoptera_acutorostrata_scammoni MEPKARCA DSQRGAPC T-NHEPP- -ALEEGGR R--HR-RV RA--GSSS GALGLQLR L--RPRPQ RR------- ---AAA-- ---AAAAA SAVLG-GR -AAAAQPI
Neophocaena_asiaeorientalis_asiaeorientalis MEPKARCA DSQRGAPC T-NHEPP- -ALEEGGR R--HR-RA RA--GSSS GALGLQLR L--RPRPQ RR------- ---AAA-- -AAAAAAA SAVLG-GR -AAAAQPI
Lagenorhynchus_obliquidens MEPKARCA DSQRGAPC T-NHEPP- -ALEEGGR R--HR-RA RA--GSSS GALGLQLR L--RPRPQ RR------- ---AAAAA AAVAAAAA SAVLG-GR -AAAAQPI
Physeter_catodon MEPKARCA DSQRGAPC T-NHEPP- -ALEEGGR R--HR-RA RA--GSSS GALGLQLR L--RPRPQ RR------- ---A---- ---AAAAA SAVLG-GR -AAAAQPI
Vicugna_pacos MESKARCP DSHRGAPC T-NHEPP- -ALEEGGR R--HR-RA RA--GSSS GALGLQLR P--RPRPQ RR------- ---AAA-- ---AAAAA SAILG-GR -AAAAQPI
Mustela_putorius_furo MEPKARCP DSQRGAPC T-NHEPP- -ALEEGGR R-HHR-RA RA--SSSS GALGLQLR P--RPRPQ RR------- ---AAA-- ---VAAAA SAILG-GR -AAAAQPI
Enhydra_lutris_kenyoni MEPKARCP DSQRGAPC T-NHEPP- -ALEEGGR R-HHR-RA RA--SSSS GALGLQLR P--RPRPQ RR------- ---AAA-- ---VAAAA SAILG-GR -AAAAQPI
Odobenus_rosmarus_divergens MEPKARCP DSQRGAPC T-NHEPP- -ALEEGGR R-HHR-RA RA--SSSS GALGLQLR P--RPRPQ RR------- ---AAA-- ---AAAAA RAILG-GR -AAAVQPV
Eumetopias_jubatus MEPKARCP DSQRGAPC T-NHEPP- -ALEEGGR R-HHR-RA RA--SSSS GALGLQLR P--RPRPQ RR------- ---AAA-- ---AAAAA RAILG-GR -AAAVQPV
Zalophus_californianus MEPKARCP DSQRGAPC T-NHEPP- -ALEEGGR R-HHR-RA RA--SSSS GALGLQLR P--RPRPQ RR------- ---AAA-- ---AAAAA RAILG-GR -AAAVQPV
Leptonychotes_weddellii MEPKARCP DSQRGAPC T-NHEPP- -ALEEGGR RHHHR-RA RASSSSSS GALGLQLR P--RPRPQ RR------- ---AAA-- ---AAAAA SAILG-GR -AAAVQPV
Felis_catus MEPKARCP DSQRGAPC T-NHEPP- -ALEEGGR R-HHR-RA RA--SSSS RALGLRLR P--RPRPQ RR------- ---AAA-- ---AAAAA SAILG-GR -AAAAQPV
Panthera_pardus MEPKARCP DSQRGAPC T-NHEPP- -ALEEGGR R-HHR-RA RA--SSSS RALGLRLR P--RPRPQ RR------- ---AAA-- ---AAAAA SAILG-GR -AAAAQPV
Acinonyx_jubatus MEPKARCP DSQRGAPC T-NHEPP- -ALEEGGR R-HHR-RA RA--SSSS RALGLRLR P--RPRPQ RR------- ---AAA-- ---AAAAA SAILG-GR -AAAAQPV
Peromyscus_maniculatus_bairdii MEPKAGCP DSQRGAPC THHHEPP- -ALEEGGQ R--QD-RP QA--GSSN RTEGVQLR RRLRPRPR RR------- -------- ---AAAVA SALFGGGR AAAAAQPA
Mesocricetus_auratus MEPKARCP DSQRGAPC T-NHEPP- -ALEEGGE P--QD-QL RA--SCSN RTQDLQLR P--RPRPQ RR------- -------- ---AAAVA NGLFG-GR -AAAAQPS
Microtus_ochrogaster MEPKARSP DPQRGAPC T-HHEPP- -ALEEGGE Q--QD-GL GT--SSSN RTQGLQLR P--RPRPQ RR------- -------- ---AAAVA SALFG-GR -AAAAQPF
Meriones_unguiculatus MEPKARCP DSQRGAPC T-NHEPP- -ALEEGGQ R--QG-RL RA--RASH GTLGLQLR P--RPRPQ RR------- -------- ---AAAVA SALLR-GQ AAAAAQPS
Rattus_norvegicus MEPKARCP DSQRGAPC T-NHEPP- -ALEEGGR R--QS-RL RA--STSN RTLGLQLR P--RPRPQ RR------- -------- ---AAAVA NALLR-EW -AAAAQPS
Mus_musculus MEPKARCP DSQRGAPC T-NHEPP- -ALEEGGW R--QG-RL RA--STSN RALGLQLR P--RPRPQ RR------- -------- ---AAAVA NALLR-EW -AAAAQPS
Mus_pahari MEPKARCP DSQRGAPC T-NHEPP- -ALEEGGW H--QG-RL RA--STSN RALGLQLR P--RLRPQ RR------- -------- ---AAAVA NALLR-EW -AAAAQPS
Mus_caroli MEPKARCP DSQRGAPC T-NHEPP- -ALEEGGW H--QG-RL RA--STSN RALGLQLR P--RPRPQ RR------- -------- ---AAAIA NALLR-EW -AAAAQPS

```
                                          105
Oryctolagus_cuniculus                     AHPDALQR AARAN-LR A-RRGDAR GGRRAPQR AAPAGARA LGAASHAL AGRGAAPT APLRGQPG PALPPPGA EAEPPLPG GGRLVVAG AAAPPAHE LGLGGGLD
Octodon_degus                             AHPDALQR AARAN-LR V-RRAGAR RGRGAPQR RALAEARA LGPGSRAL ARCGAAPA AALRGQPG PALPPPGP EAEPALPG GGQLIAAG PAAPPAPE LGLGGGLD
Cavia_porcellus                           AHPDALQR AARAN-LR V-PRAETR RGRGAPQC RALAEARA LGPARHSL ARRGAAPA AALRGQPG PALPPPGP EAEPALLG GGQLAAAG PAAPPAPE LGLGGGLD
Chinchilla_lanigera                       AHPDALEG AARAD-LR V-RRAEAR RGRGAPQR RALAEARA LGPGGRAL ARRGAAPA AALRGQPG PALPPPGP EAEPALPG GGQLAAAG PAAPPAPE LGLGGGLD
Fukomys_damarensis                        AHPDALQR AARAN-LR V-RRTEAG RGRRAPQH RALAEARA LGPAGHAL ARRGVAPA AALRGQPG PALPPPGP EAELALPG GGQLAAAG PAAPAAPE LGLGGGLD
Heterocephalus_glaber                     AHPDALQR AARAN-LR V-RRTQAR RGRRAPQC RALAEARA LGPASHAL ARRGAAPA AALRGQPG PALPPPGP EAELALPG GGRIAAAG PAAPPAPE LGLGGGLD
Castor_canadensis                         AHPDAVAR AARAN-LR A-RRGEAR RGRGAPQR GAPAEARA LGAANRPL ARRGAAPA AAQGGQPG PALPPPGP EAEHALPG GGQLAVAG PAAAQAPQ LGLGGGLD
Tupaia_chinensis                          AHPDALER AARAN-LR A-RRRAAQ RGRGAPQR RALAEARA LGAASHAL ARRGAAPA APLRGQPG PALPPPGP EAEPALPG GGQLAFAG PAAAQAPE LGLGGGLD
Myotis_lucifugus                          -------- ---AD-IR GRRRGDAR RVRGAPQR GAIAEARS LGAPGSAP ARRGAAPA APLRGQPG PALPPPGA EAEPALPG GGPLALAG PAAPQAPE LGLGGGLD
Desmodus_rotundus                         AHSDAVER ASRAN-LR A-RRGDPR RGCGAPQR RAPAEARA LGAAGRTL ARRGAAPA VPLRGRPG PALPPPGP EAEPALPG GGQLALAG PAAPQAAE LGLGGGLD
Phyllostomus_discolor                     AHPDAVER ASRAN-LR A-RRGDAQ RGRGAPQR RAPAEARA LGAAGRAL ARRGTAPA AALRGRPG PALPPPGP EAEPALPG GGQRALAG PAAPQAAE LGVGGGLD
Nannospalax_galili                        AHPDALER AARAN-LR A-RRGDAR RGRGATQR PAPTEARA LGAAGHAL ARRGAAPA PALRGQPG PALPPPGP EAEPALPG GGQLVIAG PAAAPAPE LGLGGGLD
Eptesicus_fuscus                          AHPDALEG ASRAN-LR ARRRGDAR RARGAPQR GAPAEARA LGAAGRAP ARRGAAPA APLRGQPG PALPPPGA EAEPALPG GGPLAAAG PAAPQAPE LGLGGGLD
Equus_caballus                            AHPDALQG APRAD-LR A-RRGAAR RGRGAPQR RAPAEARA LGAAGRAP ARRGAAPA APLRGQPG PALPPPGP EAEPALPG GGQLAVAG AAAPAAPE LGLGGGLD
Equus_przewalskii                         AHPDALQG APRAD-LR A-RRGAAR RGRGAPQR RAPAEARA LGAAGRAP ARRGAAPA APLRGQPG PALPPPGP EAEPALPG GGQLAVAG AAAPAAPE LGLGGGLD
Ceratotherium_simum_simum                 AHPDALQR ASRAN-LR A-RRGAAR RGRGAPQR RAPAEARA LGAASRDP ARRGAAPA APLRGQPG PALPPPGP EAEPALPG GGQLAIAG PAAPAAPH LGLGGGLD
Bison_bison_bison                         -------- ------VA A-RRGESR RGRGARQR GAPAEARA LGAAGPAL ARRGAAPA APSRGQPG PALPHPGA EAEPALPG GGQLALAG AAAPAAPE LGLGGGLD
Bos_indicus                               AHPDALER APRAD-LR X-RRGEAR RGRGAPQR GAPAEARA LGAASPAL ARRGAAPA APPRGQPG PALPHPGA EAEPALPG GGQLALAG AAAPAAPE LGLGGGLD
Bos_indicus_x_Bos_taurus                  AHPDALER APRAD-LR A-RRGEAR RGRGAPQR GAPAEARA LGAASPAL ARRGAAPA APPRGQPG PALPHPGA EAEPALPG GGQLALAG AAAPAAPE LGLGGGLD
Bos_taurus                                AHPDALER APRAD-LR A-RRGEAR RGRGAPQR GAPAEARA LGAASPAL ARRGAAPA APPRGQPG PALPHPGA EAEPALPG GGQLALAG AAAPAAPE LGLGGGLD
Odocoileus_virginianus_texanus            AHPDALER APRAD-LR A-RRGEAR RGRGAPQR GAPAEARA LGAADRAL ARRGAAPA APPRGQPG PALPPPGA EAEPALPG GGQLALAG AAAPAAPE LGLGGGLD
Capra_hircus                              AHPNALER APRAD-LR A-RRGEAR RGRGAPQR GAPAEARA LGAAGHAL ARRGAAPA APPRGQPG PALPPPGA EAEPALPG GGQLALAG AAAPAAPE LGLGGGLD
Ovis_aries                                AHPNALER APRAD-LR A-RRGEAR RGRGAPQR GAPAEARA LGAAGRAL ARRGAAPA APPRGQPG PALPPPGA EAEPALPG GGQLALAG AAAPAAPE LGLGGGLD
Ovis_aries_musimon                        AHPNALER APRAD-LR A-RRGEAR RGRGAPQR GAPAEARA LGAAGRAL ARRGAAPA APPRGQPG PALPPPGA EAEPALPG GGQLALAG AAAPAAPE LGLGGGLD
Otolemur_garnettii                        EHPDALER AARAN-LR A-RRGDAR RSRGASQR RTPAEARA LGALNRAL ARRGAAPA APLRGQPG PALPPLGP EAEPALPG GGQLAIAG PAAPAAPE LGLGGGLD
Manis_javanica                            AHPDALER APRAN-LR A-HRGGAR RGRGAPQH RAPAEARA LGAVGHAL ARRGAAPA APLWGQPG PALLPPGP EAEPALPG GGQLAIAG PAAPPAPE LGLGGGLD
Hipposideros_armiger                      AHPDALER ASRAN-LR A-RRGDAR RGRGTPQR RAPAEARA LGAAGRSL ARRGAAPP APLRGRPG PALPPPGP EAEPALPG GGQLAIAG PAAPAAPE LGLGGGLD
Dasypus_novemcinctus                      AHPDALQR AARAD-LR A-RRGDAR RGRGAPQR RAPAEARA LGAARRAL ARRGAAPA APLRGQPG PALPYPGP EAEPALPG DGQLAVAG PAAPPAPD LGLGGGLD
Canis_lupus_dingo                         AHPDALER ASRAN-LR E-RRGDAR RGRRAPQH RAPAEARA LGAAGHAL AGRGAAPA APLRGQPG PALPPPGP EAEPALPG GGQLVIAG PAAPAAPE LGLGGGLD
Canis_lupus_familiaris                    AHPDALER ASRAN-LR E-RRGDAR RGRRAPQH RAPAEARA LGAAGHAL AGRGAAPA APLRGQPG PALPPPGP EAEPALPG GGQLVIAG PAAPAAPE LGLGGGLD
Rousettus_aegyptiacus                     AHPDALER ASRAN-LR A-RRGDAR RGRGAPQR RTPAEARA LGAAGRAL ARRGAAPT PPLRGQPG PALPPPGP EAEPALPG GGQLAIAG PAAPPAPE LGLGGGLD
Pteropus_alecto                           AHPDALER ASRAN-LR A-RRGDAR RGRGAPQR RTPAEARA LGAAGRAL ARRGAAPT PPLRGQPG PALPPPGA EAEPALPG GGQLAIAG PAAPPALE LGLGGGLD
Pteropus_vampyrus                         AHPDALER ASRAN-LR A-RRGDAR RGRGAPQR RTPAEARA LGAAGRAL ARRGAAPT PPLRGQPG PALPPPGA EAEPALPG GGQLAIAG PAAPPAPE LGLGGGLD
Microcebus_murinus                        AHPDALER AARAN-LR A-RRGDAR RGRSATQR RAPAEARA LGAASRAL ARRGAAPA APLRGQPG PALPPPGP EAEPALPG GGQLAIAG PAAPAAPE LGLGGGLD
Propithecus_coquereli                     AHPDALER AARAN-LR A-RRGDAR RGRGATQH RAPAEARA LGAASRAL ARRGAAPA APLRGQPG PALPPPGP EAEPALPG GGQLAVAG PAALAAPE LGLGGGLD
Loxodonta_africana                        AHPDALER APRPD-LR P-RRGDAR RGRGAPQR RAPAEARA LGAASRVL ARRGAVPA APLRGQPG PALPPPGP EAEPALLG GGQLAIAG PAAPAAPE LGMGGGLD
Trichechus_manatus_latirostris            AHPDALER ASRPD-LR A-RRGDAR RGRGAPQH RAPAEARA LGAASRTL ARRGAVPA APLRGQPG PALPPPGP EAEPALLG GGQLAIAG PAAPPAPE LGLGGGLD
Sus_scrofa                                AHPDALER APRAN-LR A-RRGDAR RGRGAPQR RAPAEARA LGAAGLAL AGRGAAPA APLRGQPG PALPPPGP EAEPALPG GGQLPLAG PAAPPALE LGLGGGVD
Theropithecus_gelada                      AHPDALER AARAN-LR A-RRGDTR RGRGAPQR RTPAEARA LGAASSAL ARRGAAPA APLRGQPG PALPPPGP EAEPALPG GGQLAVAG PAAPAAHG LGLGGGLD
Chlorocebus_sabaeus                       AHPDALER AARAN-LR A-RRGDTR RGRGAPQR RTPAEARA LGAASSAL ARRGAAPA APLRGQPG PALPPPGP EAEPALPG GGQLAVAG PAAPAAHG LGLGGGLD
Cercocebus_atys                           AHPDALER AARAN-LR A-RRGDTR RGRGAPQR RTPAEARA LGAASSAL ARRGAAPA APLRGQPG PALPPPGP EAEPALPG GGQLAVAG PAAPAAHG LGLGGGLD
Macaca_nemestrina                         AHPDALER AARAN-LR A-RRGDTR RGRGAPQR RTPAEARA LGAASSAL ARRGAAPA APLRGQPG PALPPPGP EAEPALPG GGQLAVAG PAAPAAHG LGLGGGLD
Mandrillus_leucophaeus                    AHPDALER AARAN-LR A-RRGDTR RGRGAPQR RTPAEARA LGAASSAL ARRGAAPA APLRGQPG PALPPPGP EAEPALPG GGQLAVAG PAAPAAHG LGLGGGLD
Macaca_fascicularis                       AHPDALER AARAN-LR A-RRGDTR RGRGAPQR RTPAEARA LGAASSAL ARRGAAPA APLRGQPG PALPPPGP EAEPALPG GGQLAVAG PAAPAAHG LGLGGGLD
Papio_anubis                              AHPDALER AARAN-LR A-RRGDTR RGRGAPQR RTPAEARA LGAASSAL ARRGAAPA APLRGQPG PALPPPGA EAEPALPG GGQLAVAG PAAPAAHG LGLGGGLD
Piliocolobus_tephrosceles                 AHPDALER AARAN-LR A-RRGDTR RGRGAPQR RTPAEARA LGAASGAL ARRGAAPA APLRGQPG PALPPPGP EAEPALPG GGQLAVAG PAAPAAPG LGLGGGLD
Rhinopithecus_bieti                       AHPDALER AARAN-LR A-RRGDTR RGRGAPQR RTPAEARA LGAASGAL ARRGAAPA APLRGQPG PALPPPGP EAEPALPG GGQLAVAG PAAPAAPG LGLGGGLD
Rhinopithecus_roxellana                   AHPDALER AARAN-LR A-RRGDTR RGRGAPQR RTPAEARA LGAASGAL ARRGAAPA APLRGQPG PALPPPGP EAEPALPG GGQLAVAG PAAPAAPG LGLGGGLD
Colobus_angolensis_palliatus              AHPDALER AARAN-LR A-RRGDTR RGRGAPQR RTPAEARA LGAASGAL ARRGAAPA APLRGQPG PALPPPGP EAEPALPG GGQLAVAG PAAPAAPG LGLGGGLD
Nomascus_leucogenys                       AHPDALER AARAN-LR A-RRGDAR RGRGAPQR RAPAEARA LGAASRPL ARRGAAPA APLRGQPG PALPPPGP EAEPALPG GGQLAVAG PAAPAAPA LGLGGGLD
Pongo_abelii                              AHPDALER AARAN-LR A-RRGDAR RGRGAPQR RAPAEARA LGAASRAL ARRGAAPA APLRGQPG PALPPPGP EAEPALLG GGQLAVAG PAAPEAPG LGLGGGLD
Pan_troglodytes                           GHPDALER AARAN-LR A-RRGDAW RGRGAPQR RAPAEARA LGAASRAL ARRGAAPA APLRGQPG PALPPPGP EAEPALPG GGQLAVAG PAAPEAPG LGLGGGLD
Gorilla_gorilla_gorilla                   GHPDALER AARAN-LR A-RRGDAW RGRGAPQR RAPAEARA LGAASRAL ARRGAAPA APLRGQPG PALPPPGP EAEPALPG GGQLAVAG PAAPEAPG LGLGGGLD
Homo_sapiens                              GHPDALER AARAN-LR A-RRGDAW RGRGAPQR RAPAEARA LGAASRAL ARRGAAPA APLRGQPG PALPPPGP EAEPALPG GGQLAVAG PAAPEAPG LGLGGGLD
Pan_paniscus                              GHPDALER AARAN-LR A-RRGDAW RGRGAPQR RAPAEARA LGAASRAL ARRGAAPA APLRGQPG AALPPPGP EAEPALPG GGQLAVAG PAAPEAPG LGLGGGLD
Saimiri_boliviensis_boliviensis           AHPDALER AARAN-LR A-RRGDAR RGRGAPQR RAPAEARA LGAASRAL ARRGAAPA SPLRGQPG RALPPPGP EAEPALPG GGQLAVAG PAAPAAPE LGLGGGLD
Callithrix_jacchus                        AHPDALER AARAN-LR A-RRGDAR RGRGAPQR RAPAEARA LGAASHAL ARRGAAPA APLRGQPG RAFPPPGP EAEPALPG GGQLAVAG PAAPAAPE LGLGGGLD
Cebus_capucinus_imitator                  AYPDALER AARAN-LR A-RRGDAR RGRGAPQR RAPAEARA LGAASRAL ARRGAAPA VPLRGQPG RALPPPGP EAEPALPG GGQLAVAG PAAPAAPE LGLGGGLD
Aotus_nancymaae                           AHPDALER AARAN-LR A-RRGDAR RGRGAPQR RAPAEARA LGAASRAL ARRGAAPA APIRGQPG RALPPPGP EAEPALPG GGQLAVAG PAAPAAPE LGLGGGLD
Urocitellus_parryii                       AHPDALER AARAN-LR A-RRGDAR RGRGAPQR RASAEARA LGAAGRAH ARRGVAPA AALRGQPG PALPPPGP EAEPALPG GGQFPVAG PAAPPAPE LGLGGGLD
Marmota_marmota_marmota                   AHPDALER ASRAN-LR A-RRGDAR RGRGAPQR RASAEARA LGAAGRAR ARRGVAPA AALRGQPG PALPPPGP EAEPALPG GGQLPVAG PAAPPAPE LGLGGGLD
Marmota_flaviventris                      AHPDALER ASRAN-LR A-RRGDAR RGRGAPQR RASAEARA LGAAGRAH ARRGVAPA AALRGQPG PALPPPGP EAEPALPG GGQLPVAG PAAPPAPE LGLGGGLD
Ictidomys_tridecemlineatus                AHPDALER AARAN-LR A-RRGDAR RGRGAPQR RASAEARA LGAAGRAL ARRGAAPA AALRRQPG PALPPPGP EAELALPG GGQLPVAG PAAPPAPE LGLGGGLD
Ursus_arctos_horribilis                   AHPDAVER ASRAN-LW A-RRGDAR RGRGAPQR RAPAEARA LGAAGHAL AGRRAAPA APLRGRPG PALPPPGP EAEPALPG GGQLIIAG PAAPPAPE LGLGGGLD
Ailuropoda_melanoleuca                    AHPDAVER ASRAN-LR A-RRGDAR RGRGAPQR RAPAEARA LGAAGHAL AGRGAAPA AALRGQPG PALPPPGP EAEPALPG GGQLVIAG PAAPPAPE LGLGEGLD
Orcinus_orca                              AHPDALER ASRAN-LR A-RRGDAR RGRGAPQR RAPAEARA LGAAGRAL ARRGAAPA APLRGQPG PALPPPGP EAEPALPG GGQLVIAG PAGPPAPE LGLGGGLD
Balaenoptera_acutorostrata_scammoni       AHPDALER ASRAN-LR A-RRGDAR RGRGAPQR RAPAEARA LGSAGRAL ARRGAAPA APLRGQPG PALPPPGP EAEPALPG GGQLVIAG PAGPPAPE LGLGGGLD
```

```
Neophocaena_asiaeorientalis_asiaeorientalis AHPDALER ASRAN-LR A-RRGDAR RGRGAPQR RAPAEARA LGAGGRAL ARRGAAPA APVRGQPG PALPPPGP EAEPALPG GGQLVIAG PAGPPAPE LGLGGGLD
Lagenorhynchus_obliquidens                  AHPDALER ASRAN-LR A-RRGDAR RGRGAPQR RAPAEARA LGAAGRAL ARRGAAPA APLRGQPG PALPPPGP EAEPALPG GGQLVIAG PAGPPAPE LGLGGGLD
Physeter_catodon                            AHPDALER ASRAN-LR A-RRGDAR RGRGAPQR RAPAEARA LGAAGRAL ARRGAAPA APLRGQPG PALPPPGP EAEPALPG GGQLVIAG PAGPPAPE LGLGGGLD
Vicugna_pacos                               AHPDALER ASRAN-LR A-RRGDAR RGRGAPQR RA------ -------- -------- -------- -------- -------- -------- -------- --------
Mustela_putorius_furo                       AHPDALER ASRAN-LR A-RRGDAW RGRGAPQR RAPAAARA LGAAGHAL AGRGAAPA APLRGQPG PALPAPGP EAEPALPG GGQLVIAG PAAPPAPE LGLGGGLD
Enhydra_lutris_kenyoni                      AHPDALER ASRAN-LR A-RRGDAR RGRGAPQR RAPAAARA LGAAGHAL AGRGAAPA AALRGRPG PALPPPGP EAEPALPG GGQLVIAG PAAPQAPE LGLGGGLD
Odobenus_rosmarus_divergens                 AHPDALER ASRAN-LR A-RRGDAR RGRGAPQR RAPAEARA LGAADHAL AGRGPAPA APLRGQPG PALPPPGP EAEPALPG GGQLVIAG PAAPPAPE LGLGGGLD
Eumetopias_jubatus                          AHPDALER ASRAN-LR A-RRGDAR RGRGAPQR RAPAEARA LGAADHAL AGRGPAPA APLRGQPG PALPPPGP EAEPALPG GGQLVIAG PAAPPAPE LGLGGGLD
Zalophus_californianus                      AHPDALER ASRAN-LR A-RRGDAR RGRGAPQR RAPAEARA LGAADHAL AGRGAAPA APLRGQPG PALPPPGP EAEPALPG GGQLVIAG PAAPPAPE LGLGGGLD
Leptonychotes_weddellii                     AHPDALER ASRAN-LR A-RRGDAR RGRGAPQR RAPTGARA LGAAGHAL AGRGAAPA APLRGQPG PALPPPGP EAEPALPG GGQLVIAG PAAPPAPE LGLGGGLD
Felis_catus                                 AHPDALER ASRAN-LR A-RRGEAR RGRGAPQR RAPAEARA LGAAGHAL AGRGAAPA APLRGQPG PALPPPGP EAEPALPG GGQLVIAG PAAPPAPE LGLGGGLD
Panthera_pardus                             AHPDALER ASRAN-LR A-RRGEAR RGRGAPQR RAPAEARA LGAAGHAL AGRGAAPA APLRGQPG PALPPPGP EAEPALPG GGQLVIAG PAAPPAPE LGLGGGLD
Acinonyx_jubatus                            AHPDALER ASRAN-LR A-RRGEAR RGRGAPQR RAPAEARA LGAAGHAL AGRGAAPA APLRGQPG PALPPPGP EAEPALPG GGQLVIAG PAAPPAPE LGLGGGLD
Peromyscus_maniculatus_bairdii              AHPDAVEG AARANLLR V-RRGAPR RGRGAAQH PAPAEARA LGAAGHAH ARRAAEPA PAPGGQPG PALPPPGP EAEHALLG GGRLVIGG PVAAPARE VGLGGGLD
Mesocricetus_auratus                        AHPDALER AARAN-LR V-RRGEAR RGRGAAQH QAPAEARA LGAADHAL ARRAAEPA PAPRGQPG PALPPPGP EAEPALLG GGRLFIGG PIAAPARE LGLGGGLD
Microtus_ochrogaster                        AHPDAVER AARAN-LR V-RRGEAR RGRGAAQH QAPAEARA LGAAGHAL ARRAAAPA PALRGQPG PALPPPGP EAEPALLG GGRLIIRG PIAAPAQE LGLGGGLD
Meriones_unguiculatus                       AHPDAVER AARAN-LR V-RRGSPR RGRGTPQH RAPAEARA LGAAVHAL ARRGAAPA PALRGRPR PALPPRGP EAEPALLG GGRLVIGD PFAAPAPE LGLGGGLD
Rattus_norvegicus                           AHPDAVER PARAN-LR V-RRGRAR RGRGAAQH RAPAEARA LGAADHAL ARRAAAPA PALRGQPG PALPPPGP EAEPPVLG GGRLVIGG PIAPPAQE VGLGGGLD
Mus_musculus                                AHPDVVER PARAD-LR V-RRGNAR RGRGAAQR RAPAEARA LGAAGHAL ARRGAAPA PALRGQPG PALPPPGP EAEPALLG GGRLVIGG PIAPRAQE LGLGGGLD
Mus_pahari                                  AHPDAVER PARAN-LR V-RRGNAR RGRGAAQH RAPAEARA LGAAGHAL ARRGAAPA PALRGQPG PALPPPGP EAEPALLG GGRLVIGG PIAPRAQE LGLGGGLD
Mus_caroli                                  AHPDAVER PARAD-LR V-RRGNAR RGRGAAQH RAPAEARA LGAAGHAL ARRGAAPA PALGGQPG PALPPPGP EAEPALLG GGRLVIGG PIAPRAQE LGLGGGLD
```

```
                                   209
Oryctolagus_cuniculus              PVRPRGGA RPRGRPRG AGPGVRRG GLLGRGNL PHAGGGHI PLGLVFL- --VQPAAG GRALLLDQ PAVAG
Octodon_degus                      AVRPRGGG RTRGHPRG AGPGVRRG GLLGRGNL PHVGGGHI PLGLVFL- --VQPAAG GRALLLDQ PAVAG
Cavia_porcellus                    AVRPRGGG RTRGHPRG AGPGVRRG GLLGRGNL PHIGGGHI PLGLVFL- --VQPAAG GRALLLDQ PAVAG
Chinchilla_lanigera                AVRPRGGG RTRGHPRG AGPGVRRG GLRGRGNL PHIGGGHI PLGLVFL- --VQPAAG GRALLLDQ PAVAG
Fukomys_damarensis                 AVRPRGGG RTRGHPRG AGPGVRRG GLLGRGNL PHVGGGHI PLGLVFL- --VQPAAG GRALLLDQ PAVAG
Heterocephalus_glaber              AVRPRGGG RTRGHPRG AGPGVRRG GLLGRGNL PHIGGGHI PLGLVFL- --VQPAAG GRALLLDQ PAVAG
Castor_canadensis                  PVRPRGGG RTRGHPRG AGPGVRRG GLLGRGNL PHIGGGHI ALGLVFL- --VQPAAG GRALLLDQ PAVAG
Tupaia_chinensis                   PVRPRGGG RTRGHPRG AGPGVRRG GLLGRGNL PHVGGGHI PLGLVFL- --VQPAAG GRALLLDQ PAVAG
Myotis_lucifugus                   PVRPRGGG RTRGHPRG AGPGVRRG GLLGRGNL PHAGGGHI PLGLVFL- --VQPAAG GRALLLDQ PAVAG
Desmodus_rotundus                  PVRPRGGG RTRGHPRG AGPGVRRG GLLGRGNV PHAGGGHI PLGLVFL- --VQPAAG GRALLLDQ PAVAG
Phyllostomus_discolor              PVRPRGGG RTRGHPRG AGPGVRRG GLLGRGNV PHAGGGHI PLGLVFL- --VQPAAG GRALLLDQ PAVAG
Nannospalax_galili                 PLRPRGGG RTRGHPRG AGPGVRRG GLLGRGNL PHIGGGHI PLGLVFL- --VQPAAG GRALLLDQ PAVAG
Eptesicus_fuscus                   PVRPRGGG RTRGHPRG AGPGVRRG GLLGRGNL PHAGGGHI PLGLLLAG LSLADCPG PRSQ---- -----
Equus_caballus                     PVRPRGGG RTRGHPRG AGPGVRRG GLLGRGNL PHVGGGHI PLGLVFL- --VQPAAG GRALLLDQ PAVAG
Equus_przewalskii                  PVRPRGGG RTRGHPRG AGPGVRRG GLLGRGNL PHVGGGHI PLGLVFL- --VQPAAG GRALLLDQ PAVAG
Ceratotherium_simum_simum          PVRPRGGG RTRGHPRG AGPGVRRG GLLGRGNL PHVGGGHI PLGLVFL- --VQPAAG GRALLLDQ PAVAG
Bison_bison_bison                  PVRPRGGG RTRGHPRG AGPGVRRG GLLGRGNL PHVGGGHI PLGLVFL- --VQPAAG GRALLLDQ PAVAG
Bos_indicus                        PVRPRXGG RTRGHPRG AGPGVRRG GLLGRGNL PHVGGGHI PLGLVFL- --VQPAAG GRALLLDQ PAVAG
Bos_indicus_x_Bos_taurus           PVRPRGGG RTRGHPRG AGPGVRRG GLLGRGNL PHVGGGHI PLGLVFL- --VQPAAG GRALLLDQ PAVAG
Bos_taurus                         PVRPRGGG RTRGHPRG AGPGVRRG GLLGRGNL PHVGGGHI PLGLVFL- --VQPAAG GRALLLDQ PAVAG
Odocoileus_virginianus_texanus     PVRPRGGG RTRGHPRG AGPGVRRG GLLGRGNL PHAGGGHI PLGLVFL- --VQPAAG GRALLLDQ PAVAG
Capra_hircus                       PVRPRGGG RTRGHPRG AGPGVRRG GLLGRGNL PHVGGGHI PLGLVFL- --VQPAAG GRALLLDQ PAVAG
Ovis_aries                         PVRPRGGG RTRGHPRG AGPGVRRG GLLGRGNL PHVGGGHI PLGLVFL- --VQPAAG GRALLLDQ PAVAG
Ovis_aries_musimon                 PVRPRGGG RTRGHPRG AGPGVRRG GLLGRGNL PHVGGGHI PLGLVFL- --VQPAAG GRALLLDQ PAVAG
Otolemur_garnettii                 PVRPRGGG RTRGHPRG AGPGVRRG GLLGRGNL PHIGGGHI PLGLVFL- --VQPAAG GRALLLDQ PAVAG
Manis_javanica                     PVRPRGGG RTRGHPRG AGPGVRRG GLLGRGNL PHIGGGHI PLGLVFL- --VQPAAG GRALLLDQ PAVAG
Hipposideros_armiger               PVRPRGGG RTRGHPRG AGPGVRRG GVLGRGNL PHAGGGHI PLGLVFL- --VQPAAG GRALLLDQ PAIAG
Dasypus_novemcinctus               AVRPRGGG RTRGHPRG AGPGVRRG GLLGRGNL PHVGGGHI PLGLVFL- --VQPAAG GRALLLDQ PAVAG
Canis_lupus_dingo                  PVRPRGGG RTRGHPRG AGPGVRRG GLLGRGNL PHIGGGHI PLGLVFL- --VQPAAG GRALLLDQ PAVAG
Canis_lupus_familiaris             PVRPRGGG RTRGHPRG AGPGVRRG GLLGRGNL PHIGGGHI PLGLVFL- --VQPAAG GRALLLDQ PAVAG
Rousettus_aegyptiacus              PVRPRGGG RTRGHPRG AGPGVRRG GLLGRGNL PHAGGGHI PLGLVFL- --VQPAAG GRALLLDQ PAVAG
Pteropus_alecto                    PVRPRGGG RTRGHPRG AGPGVRRG GLLGRGNL PHAGGGHI PLGLVFL- -------- -------- -----
Pteropus_vampyrus                  PVRPRGGG RTRGHPRG AGPGVRRG GLLGRGNL PHAGGGHI PLGLVFL- --VQPAAG GRALLLDQ PAVAG
Microcebus_murinus                 AVRPRGGG RTRGHPRG AGPGVRRG GLLGRGNL PHVGGGHI PLGLVFL- --VQPAAG GRALLLDQ PAVAG
Propithecus_coquereli              PVRPRGGG RTRGHPRG AGPGVRRG GLLGRGNL PHIGGGHI PLGLVFL- --VQPAAG GRALLLDQ PAVAG
Loxodonta_africana                 PVRPRGGG RTRGHPRG AGPGVRRG GLLGRGNL PHIGGGHI PLGLVFL- --VQPAAG GRALLLDQ PAIAG
Trichechus_manatus_latirostris     PVRPRGGG RTRGHPRG AGPGVRRG GLLGRGNL PHVGGGHI PLGLVFL- --VQPAAG GRALLLDQ PAIAG
Sus_scrofa                         PVRPRGGG RTRGHPRG AGPGVRRG GLLGRGNL PHIGGGHI PLGLVFL- --VQPAAG GRALLLDQ PAVAG
Theropithecus_gelada               PVRPRGGG RTRGHPRG AGPGVRRG GLLGRGNL PHIGGGHI PLGLVFL- --VQPAAG GRALLLDQ PAVAG
Chlorocebus_sabaeus                PVRPRGGG RTRGHPRG AGPGVRRG GLLGRGNL PHIGGGHI PLGLVFL- --VQPAAG GRALLLDQ PAVAG
Cercocebus_atys                    PVRPRGGG RTRGHPRG AGPGVRRG GLLGRGNL PHIGGGHI PLGLVFL- --VQPAAG GRALLLDQ PAVAG
Macaca_nemestrina                  PVRPRGGG RTRGHPRG AGPGVRRG GLLGRGNL PHIGGGHI PLGLVFL- --VQPAAG GRALLLDQ PAVAG
Mandrillus_leucophaeus             PVRPRGGG RTRGHPRG AGPGVRRG GLLGRGNL PHIGGGHI PLGLVFL- --VQPAAG GRALLLDQ PAVAG
Macaca_fascicularis                PVRPRGGG RTRGHPRG AGPGVRRG GLLGRGNL PHIGGGHI PLGLVFL- --VQPAAG GRALLLDQ PAVAG
Papio_anubis                       PVRPRGGG RTRGHPRG AGPGVRRG GLLGRGNL PHIGGGHI PLGLVFL- --VQPAAG GRALLLDQ PAVAG
Piliocolobus_tephrosceles          PVRPRGGG RTRGHPRG AGPGVRRG GLLGRGNL PHIGGGHI PLGLVFL- --VQPAAG GRALLLDQ PAVAG
Rhinopithecus_bieti                PVRPRGGG RTRGHPRG AGPGVRRG GLLGRGNL PHIGGGHI PLGLVFL- --VQPAAG GRALLLDQ PAVAG
Rhinopithecus_roxellana            PVRPRGGG RTRGHPRG AGPGVRRG GLLGRGNL PHIGGGHI PLGLVFL- --VQPAAG GRALLLDQ PAVAG
Colobus_angolensis_palliatus       PVRPRGGG RTRGHPRG AGPGVRRG GLLGRGNL PHIGGGHI PLGLVFL- --VQPAAG GRALLLDQ PAVAG
Nomascus_leucogenys                PLRPRGGG RTRGHPRG AGPGVRRG GLLGRGNL PHIGGGHI PLGLVFL- --VQPAAG GRALLLDQ PAVAG
Pongo_abelii                       PVRPRGGG RTRGHPRG AGPGVRRG GLLGRGNL PHIGGGHI PLGLVFL- --VQPAAG GRALLLDQ PAVAG
Pan_troglodytes                    PVRPRGGG RTRGHPRG AGPGVRRG GLLGRGNL PHIGGGHI PLGLVFL- --VQPAAG GRALLLDQ PAVAG
Gorilla_gorilla_gorilla            PVRPRGGG RTRGHPRG AGPGVRRG GLLGRGNL PHIGGGHI PLGLVFL- --VQPAAG GRALLLDQ PAVAG
Homo_sapiens                       PVRPRGGG RTRGHPRG AGPGVRRG GLLGRGNL PHIGGGHI PLGLVFL- --VQPAAG GRALLLDQ PAVAG
Pan_paniscus                       PVRPRGGG RTRGHPRG AGPGVRRG GLLGRGNL PHIGGGHI PLGLVFL- --VQPAAG GRALLLDQ PAVAG
Saimiri_boliviensis_boliviensis    PVRPRGGG RTRGHPRG AGPGVRRG GLLGRGNL PHIGGGHI PLGLVFL- --VQPAAG GRALLLDQ PAVAG
Callithrix_jacchus                 PVRPRGGG RTRGHPRG AGPGVRRG GLLGRGNL PHIGGGHI PLGLVFL- --VQPAAG GRALLLDQ PAVAG
Cebus_capucinus_imitator           PVRPRGGG RTRGHPRG AGPGVRRG GLLGRGNL PHIGGGHI PLGLVFL- --VQPAAG GRALLLDQ PAVAG
Aotus_nancymaae                    PVRPRGGG RTRGHPRG AGPGVRRG GLLGRGNL PHIGGGHI PLGLVFL- --VQPAAG GRALLLDQ PAVAG
Urocitellus_parryii                PVRPRGGG RTRGHPRG AGPGVRRG GLLGRGNL PHIGGGHI PLGLVFL- --VQPAAG GRALLLDQ PAVAG
Marmota_marmota_marmota            PVRPRGGG RTRGHPRG AGPGVRRG GLLGRGNL PHIGGGHI PLGLVFL- --VQPAAG GRALLLDQ PAVAG
Marmota_flaviventris               PVRPRGGG RTRGHPRG AGPGVRRG GLLGRGNL PHIGGGHI PLGLVFL- --VQPAAG GRALLLDQ PAVAG
Ictidomys_tridecemlineatus         PVRPRGGG RTRGHPRG AGPGVRRG GLLGRGNL PHIGGGHI PLGLVFL- --VQPAAG GRALLLDQ PAVAG
Ursus_arctos_horribilis            PVRPRGGG RTRGHPRG AGPGVRRG GLLGRGNL PHVGGGHI PLGLVFL- --VQPAAG GRALLLDQ PAVAG
Ailuropoda_melanoleuca             PVRPRGGG RTRGHPRG AGPGVRRG GLLGRGNL PHIGGGHI PLGLVFL- --VQPAAG GRALLLDQ PAVAG
Orcinus_orca                       PVRPRGGG RTRGHPRG AGPGVRRG GLLGRGNL PHIGGGHI PLGLVFL- --VQPAAG GRALLLDQ PAVAG
Balaenoptera_acutorostrata_scammoni PVRPRGGG RTRGHPRG AGPGVRRG GLLGRGNL PHIGGGHI PLGLVFL- --VQPAAG GRALLLDQ PAVAG
```

```
Neophocaena_asiaeorientalis_asiaeorientalis  PVRPRGGG RTRGHPRG AGPGVRRG GLLGRGNL PHIGGGHI PLGLVFL- --VQPAAG GRALLLDQ PAVAG
Lagenorhynchus_obliquidens                   PVRPRGGG RTRGHPRG AGPGVRRG GLLGRGNL PHIGGGHI PLGLVFL- --VQPAAG GRALLLDQ PAVAG
Physeter_catodon                             PVRPRGGG RTRGHPRG AGPGVRRG GLLGRGNL PHIGGGHI PLGLVFL- --VQPAAG GRALLLDQ PAVAG
Vicugna_pacos                                ---PRG-G RTRGHPRG AGPGVRRG GLLGRGNL PHVGGGHI PLGLVFL- --VQPAAG GRALLLDQ PAVAG
Mustela_putorius_furo                        PVRPRGGG RTRGHPRG AGPGVRRG GLLGRGNL PHVGGGHI PLGLVFL- --VQPAAG GRALLLDQ PAVAG
Enhydra_lutris_kenyoni                       PVRPRGGG RTRGHPRG AGPGVRRG GLLGRGNL PHVGGGHI PLGLVFL- --VQPAAG GRALLLDQ PAVAG
Odobenus_rosmarus_divergens                  PVRPRGGG RTRGHPRG AGPGVRRG GLLGRGNL PHIGGGHI PLGLVFL- --VQPAAG GRALLLDQ PAVAG
Eumetopias_jubatus                           PVRPRGGG RTRGHPRG AGPGVRRG GLLGRGNL PHIGGGHI PLGLVFL- --VQPAAG GRALLLDQ PAVAG
Zalophus_californianus                       PVRPRGGG RTRGHPRG AGPGVRRG GLLGRGNL PHIGGGHI PLGLVFL- --VQPAAG GRALLLDQ PAVAG
Leptonychotes_weddellii                      PVRPRGGG RTRGHPRG AGPGVRRG GLLGRGNL PHIGGGHI PLGLVFL- --VQPAAG GRALLLDQ PAVAG
Felis_catus                                  PVRPRGGG RTRGHPRG AGPGVRRG GLLGRGNL PHAGGGHI PLGLVFL- --VQPAAG GRALLLDQ PAVAG
Panthera_pardus                              PVRPRGGG RTRGHPRG AGPGVRRG GLLGRGNL PHAGGGHI PLGLVFL- --VQPAAG GRALLLDQ PAVAG
Acinonyx_jubatus                             PVRPRGGG RTRGHPRG AGPGVRRG GLLGRGNL PHAGGGHI PLGLVFL- --VQPAAG GRALLLDQ PAVAG
Peromyscus_maniculatus_bairdii               PVRPRGGG RTRGHPRG AGPGVRRG GLPVRGNV PHFGAGHI PLGLVFL- --VQPAAG GRAVLLDQ PAIAG
Mesocricetus_auratus                         PVRPRGGG RTRGHPRG AGPGVRRG GLHGRGNL PHLGGGHI PLGLVFL- --VQPAAG GRALLLDQ PAIAG
Microtus_ochrogaster                         PVRPRGGG RTRGHPRG AGPGVRRG GLLGRGNL PHFGGGHI PLGLVFL- --VQPAAG GRALLLDQ PAIAG
Meriones_unguiculatus                        PVRPRGGG RTRGHPRG AGPGVRRG GLLGRGNL PHFGGGHI PLGLVFL- --VQPAAG GRALLLDQ PAIAG
Rattus_norvegicus                            PVRPRGGG RTRGHPRG AGPGVRRG GLLGRGNL PHFGGGHI PLGLVFL- --VQPAAG GRALLLDQ PAVAG
Mus_musculus                                 PVRPRGGG RTRGHPRG AGPGVRRG GLLGRGNL PHFGGGHI PLGLVFL- --VQPAAG GRALLLDQ PAIAG
Mus_pahari                                   PVRPRGGG RTRGHPRG AGPGVRRG GLLGRGNL PHFGGGHI PLGLVFL- --VQPAAG GRALLLDQ PAIAG
Mus_caroli                                   PVRPRGGG RTRGHPRG AGPGVRRG GLLGRGNL PHFGGGHI PLGLVFL- --VQPAAG GRALLLDQ PAIAG
```
