# Supplementary figures and images for: Evidence for a novel overlapping coding sequence in POLG initiated at a CUG start codon
Source: BMC Genet. 2020 Mar 6;21:25. doi: 10.1186/s12863-020-0828-7 (PMC7059407; doi:10.1186/s12863-020-0828-7)

Supplementary Figure 7

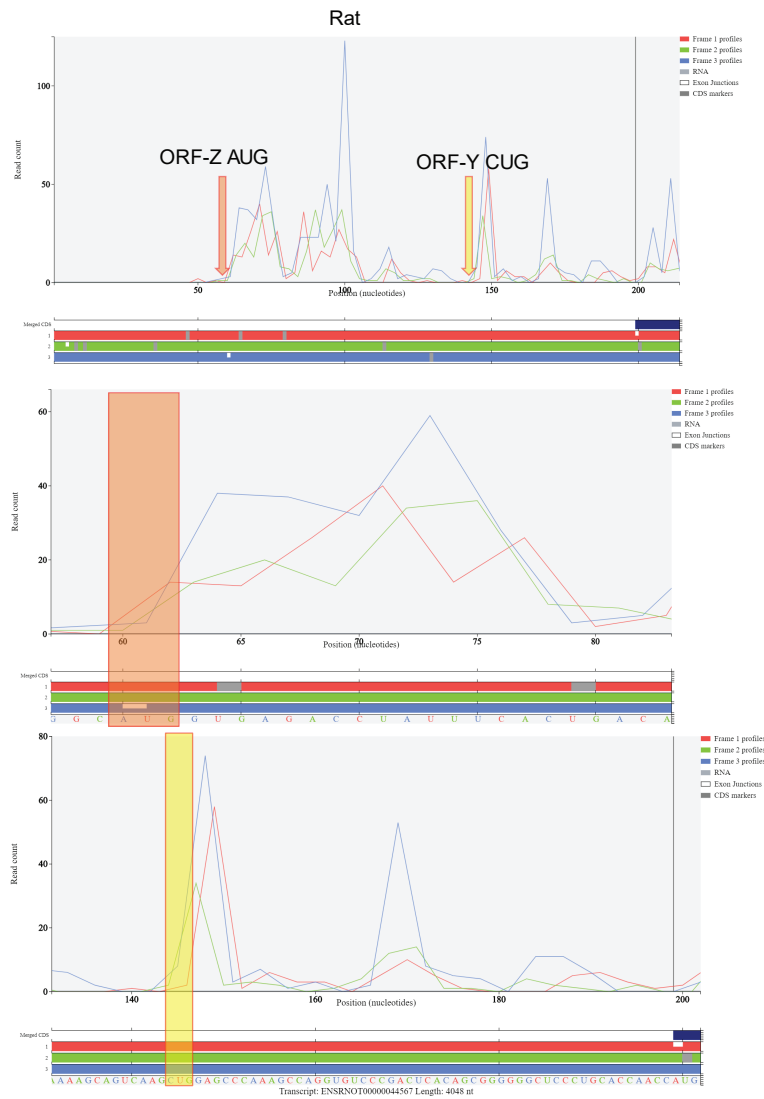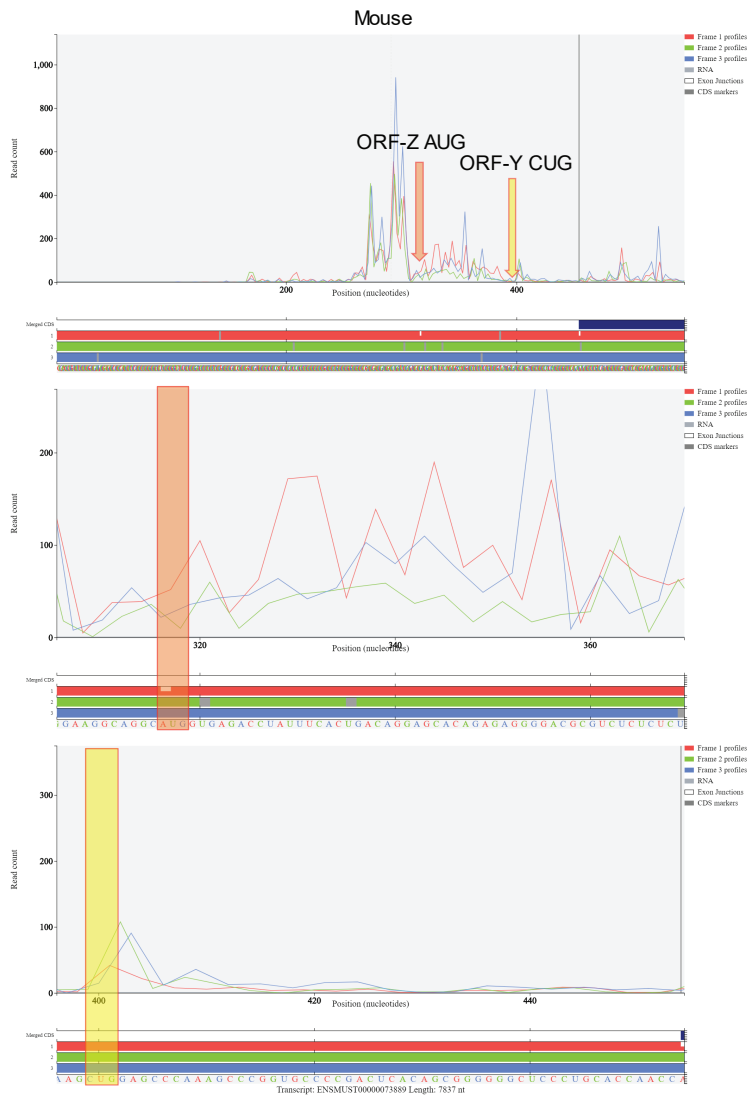

Supplement: Supplementary file 7 — Additional file 7: Figure S7 Ribosome profiling data from both Mus musculus and Rattus norvegicus mined from Trips-Viz. The red arrow and box indicate the location of the AUG for ORF-Z and the yellow arrow and box indicate the location of the CUG for ORF-Y. [file 12863_2020_828_MOESM7_ESM.pdf]
