# Supplementary material for: Evidence for a novel overlapping coding sequence in POLG initiated at a CUG start codon
Source: BMC Genet. 2020 Mar 6;21:25. doi: 10.1186/s12863-020-0828-7 (PMC7059407; doi:10.1186/s12863-020-0828-7)

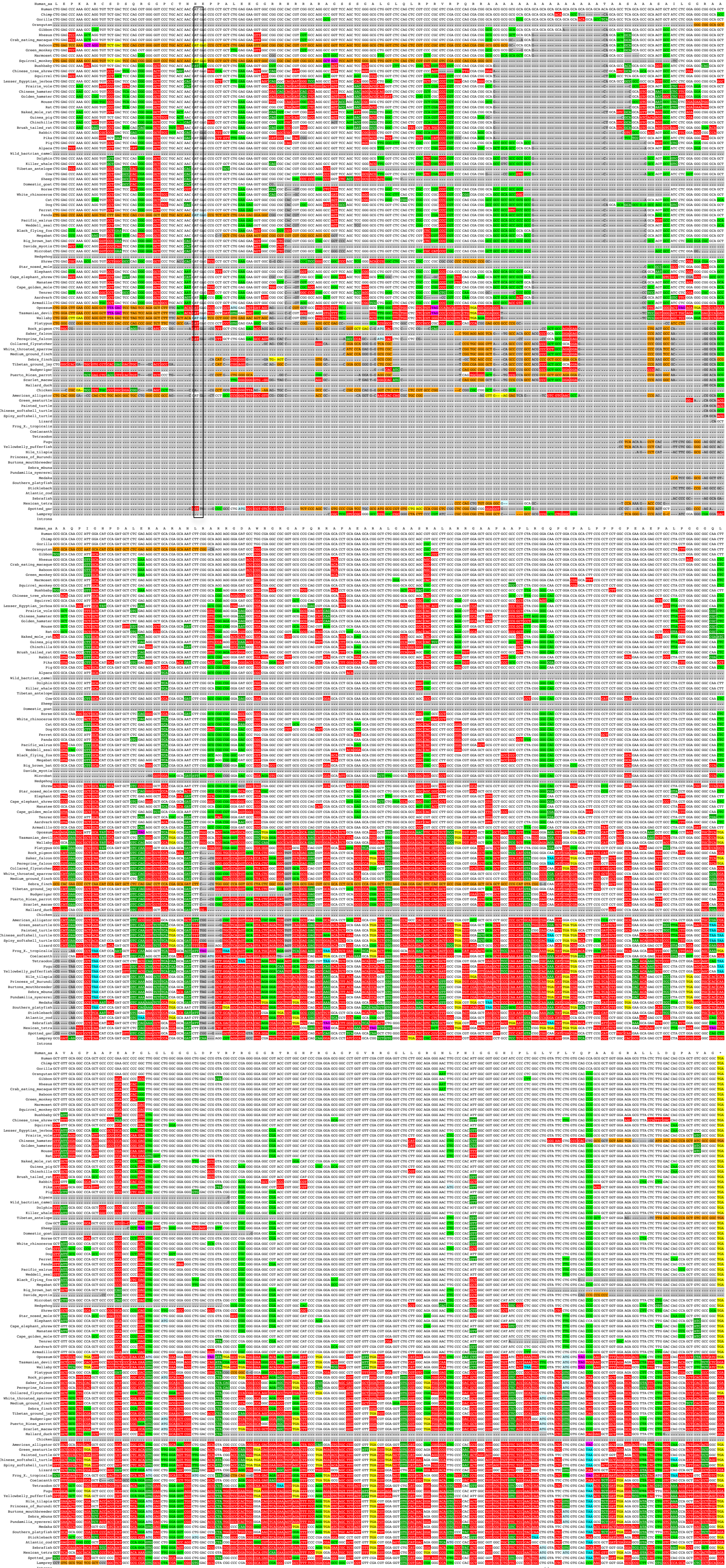

Supplement: Supplementary file 3 — Additional file 3: Figure S3 Vertebrate CodAlignView of ORF-Y. Alignment of ORF-Y sequences from 100 vertebrates, color coded using CodAlignView (see legend from Supplementary Figure 1). Insertions relative to the human sequence are not shown. The presence of frame-shifting indels and in-frame stop codons show that ORF-Y is not conserved beyond placental mammals. [file 12863_2020_828_MOESM3_ESM.pdf]
